# The Yeniseian and Andronovo Substrates in the Gene Pools of the Indigenous Southern Siberian Populations Analyzed by Q and R1a Y-Haplogroups

**DOI:** 10.3390/genes17080896

**Published:** 2026-07-30

**Authors:** Dmitry Adamov, Georgy Ponomarev, Larissa Damba, Elena Balanovska

**Affiliations:** 1 Research Centre for Medical Genetics, Moskvorechie Str. 1, Moscow 115522, Russia; pg@med-gen.ru (G.P.); larissa_damba@mail.ru (L.D.); balanovska@mail.ru (E.B.); 2 Research Institute of Medical and Social Problems and Management of the Republic of Tuva, Ulug-Khem Str. 17, Kyzyl 667003, Russia

**Keywords:** Tuvans, Scythians, Y-chromosome, haplogroups, Y-STRs, TMRCA

## Abstract

**Background/Objectives**: Due to advances in paleogenetic research, a substantial amount of data has been accumulated on the Y-chromosome of the ancient Scytho-Siberian population that inhabited South Siberia and the adjacent territories of modern Mongolia and North-West China. It became possible to compare the Y-chromosomes of ancient men and modern Tuvans of various clans. **Methods**: We analyzed Y-SNPs and Y-STRs of ancient and modern samples. **Results**: During Tuvan ethnogenesis, the total frequency of the haplogroups Q and R1a dropped from 85% in Scytho-Siberian populations to 35% in modern Tuvans. Today, the gene pool of Tuvans is dominated by haplogroups N-P43, N-M178 and O-M175, which remain undetected in the samples from the Scytho-Siberian period. An analysis of the Y-STR phylogenetic networks that integrated data on Y-STR haplotypes and Y-SNPs of the Q and R1a haplogroup branches of both ancient and modern specimens provides findings that should be interpreted as broad phylogenetic affinities consistent with the paternal lineage continuity of haplogroups Q-YP1102 and R1a-Y39884. At the same time, the TMRCA estimate for the Y-STR cluster of the dominant branch of haplogroup Q in modern Tuvans, Q-YP1102-YP1691, is only 670 ± 130 YBP. Clusters of Y-STR haplotypes of the R1a-Y39884, R1a-YP1506, and R1a-YP1543 branches in modern Tuvans are also young and date back to between 1200 and 400 YBP. The Scytho-Siberian haplogroups R1a-YP1272, Q-BZ180, and N-Y147969 were not found in our sample set of modern Tuvans (*n* = 633). **Conclusions**: Haplogroups Q-YP1102 and R1a-Y39884 originated in Siberia and their branches are typical for the modern indigenous populations of South and West Siberia. Q-YP1102 marks the Yeniseian substrate and R1a-Y39884 was common in the ancient Siberian Andronovo culture. Overall, the gene pool of the Tuvan population was shaped (at least since the Scytho-Siberian period) by close interactions with the gene pools of Siberia and West Mongolia. However, the resolution of Y-SNP and Y-STR genotyping, as well as the limited sample size of ancient samples and their location in a limited area of the Eerbek River valley, do not allow us to conclude about the direct inheritance of modern Tuvan lineages from the ancient Scytho-Siberian population.

## 1. Introduction

The area inhabited by the indigenous southern Siberian population lies at the crossroads of major ancient migration routes that ran across the forest-steppe and steppe zones of North Eurasia. From the end of the 1st millennium BCE, South Siberia was sequentially controlled by the Xiongnu Empire, the Göktürk, Uyghur and Yenisei Kyrgyz Khaganates, the Genghisid Mongol Empire, and the Manchurian Qing Empire. Today, the indigenous population of South Siberia is represented by only Turkic-speaking groups. Of them, two-thirds are Tuvans (295,000 including Todzha Tuvans), who, due to their population size, will be the major focus of this article. The rest are Altaians (78,000), who are subdivided into Northern Altaians (Kumandins, Tubalars and Chelkans) and Southern Altaians (Altai-Kizhi, Telengits and Teleuts), Khakass (61,000) represented by Kachins, Koibals, Kyzyls and Sagais, Shors (11,000), and Tofalars (700).

Importantly, the Tuvan community is still organized in clans [1,2,3], which facilitates the reconstruction of the Tuvan Y gene pool [4,5,6,7,8,9]. Although clan names and first names were transposed in the passports that Tuvans received after Tuva became part of the USSR in 1944, it is still possible to reconstruct clan affiliations from genealogies and informant evidence [9].

The gene pool of the indigenous southern Siberian population has been described using the analysis of Y-SNP and Y-STR markers [6,7,8,9,10,11,12,13,14,15,16,17,18,19,20,21,22,23,24,25,26,27,28,29,30,31,32,33,34,35,36,37,38,39]. The greatest contribution is made by haplogroup R1a-M198, which constitutes up to 60% of Southern Altaian and Shorian gene pools. There is indirect evidence that its high frequencies might reflect the genetic heritage of the Andronovo and Tashtyk archeological cultures [40]. Haplogroup R1a dominated the paternal lineages of the population of the Andronovo culture and the following Tagar and Tashtyk cultures in Southern Siberia, that is, for 2200 years, from the 18th century BCE to the 4th century CE [40].

In the context of the subject of this study, we define the term “substrate” as the ancient genetic base preserved in the form of certain branches of Y-chromosome haplogroups in the gene pool of modern populations. The “Yeniseian” substrate reflects the contribution of the paternal lineages of ancient people who spoke Yeniseian languages, while the “Andronovo” substrate represents the surviving paternal lineages of populations that left behind sites of the Andronovo archeological culture. Since the study is based exclusively on paternal lineages, it would be appropriate to emphasize that these definitions allow us to form plausible demographic scenarios consistent with genetic evidence, rather than definitive historical conclusions.

The second most common haplogroup in South Siberia is Q-M242: its highest frequencies are observed in modern Kets (~94%), Selkups (~66%) and Chelkans of the North Altai region (~50%). Some researchers have hypothesized an association between haplogroup Q-YP1102 and the Yeniseian substrate in the gene pool of the indigenous Siberian population [33,41]. Their hypothesis is corroborated by whole-genome ancient DNA evidence [42] that links ancient Yeniseian speakers to the Late Neolithic and Bronze Age populations of the Cis-Baikal region (5100–3700 YBP). Haplogroup Q-YP1691 is widespread in the modern Yeniseian, Samoyedic and Turkic populations of Siberia, including Kets, Selkups and Tuvans [42,43,44,45]; its carriers are descended from the carriers of the ancestral haplogroup Q-YP1102 from the Cis-Baikalian Neolithic Glazkovo culture.

Multiple studies of the ancient and modern northern Eurasian populations have convincingly demonstrated that the dispersal of Uralic-speaking peoples is linked to the Neolithic transcontinental migration associated with the Ymyiakhtakh archeological culture of Yakutia (~4000 YBP [42]). This massive east-to-west migration across the Eurasian forest-steppe zone was closely associated with the Seima-Turbino culture known for its unique finest bronze artifacts. In the gene pool of the migrants, the dominant haplogroup was macrohaplogroup N [42], common for most modern Finno-Ugric and Samoyedic populations. In the Tuvan gene pool, the trace of this migration reveals itself in several N-P43 branches [34,39].

Considering the above, this study will focus on the phylogeography analysis of branches of macrohaplogroups R1a, Q and, to some extent, N that connect the modern population of South Siberia to the ancient population of North Eurasia.

There is ongoing debate about the role of Mongol tribes in Tuvan ethnogenesis. The contribution of the Mongols was expected to be greater than it actually is because the Tuvans had long maintained close ethnocultural and economic contacts with the Mongol tribes from Central Asia. Of all southern Siberian ethnic groups, Tuvans have the most pronounced Mongolic features, and of all Turkic languages, the Tuvan language has the highest number of Mongolian-origin words [9,38]. However, the contribution of the Mongols to the Tuvan gene pool is minor, quite recent, and observed only in the South-East of Tuva [6,7,8,9,31,33,34,36,38]. It is hypothesized that the ancestor C-M48-SK1066 carrier population migrated to West Mongolia and Tuva in the 11th–12th centuries CE at the earliest [35].

Due to advances in paleogenetic research, there is now a large enough dataset of ancient Y-SNP and a smaller dataset of ancient Y-STR polymorphisms from the regions of Tuva and West Mongolia [46,47,48,49,50,51,52,53]. The majority of the collected ancient samples represent the Scytho-Siberian period (8th–3rd centuries BCE). At that time, the ancient population was not very diverse genetically; studies suggest that its gene pool was dominated by macrohaplogroups R1a and Q [47,48,49,50]. This assumption is supported by individual whole-genome evidence from the elite burials discovered in the region. For instance, the sample found in Tuva and assigned to the Aldy-Bel culture (7th–6th centuries BCE) belonged to haplogroup R1a-Z93-YP1456 [46], while a noble warrior sample from Chinge-Tey I (7th–6th centuries BCE) revealed haplogroup Q1b1 (Q-L476) [52].

The accumulated data allow us to raise a question regarding the nature and degree of genetic continuity between the ancient and modern populations of South Siberia and Tuva, as well as the differences between clans of modern Tuvans in the context of Y-SNP and Y-STR polymorphisms of haplogroups Q and R1a. The aim of our study is to shed light on these issues.

## 2. Materials and Methods

DNA samples of the indigenous Siberian populations (*n* = 2280: 352 Altaians, 261 Khakass, 186 Khants and Mansi, 19 Nenets, 85 Shors, 691 Siberian Tatars, 53 Tofalars, 633 Tuvans) were collected during field work supervised by Prof. Balanovska E.V. Only DNA samples from unrelated men whose ethnic affiliation was confirmed across all lines of descent for at least three generations were included in the study. Written informed consent was obtained from all donors. The study was approved by the Ethics Committee of the Research Center for Medical Genetics (Protocol № 1 dd 29 June 2020). All samples were anonymized. DNA was extracted by phenol–chloroform extraction.

The samples of the indigenous Siberian populations (*n* = 2280) were genotyped for 60 Y-SNP markers typical for the population of North Eurasia, including C-M217, C-F3791, C-F5481, C-F3918, C-M48, C-SK1066, C-M407, D-M174, E-M35, E-M78, G-M201, G1-M285, G2-P15, G2-FGC595, G2-M406, G2-P303, H-M69, I-M170, I-M253, I-P37.2, I-M223, J1-M267, J1-P58, J2-M172, J2-M12, J2-M67, J2-M92, L-M20, L-M317, N-M231, N-M128, N-Y3205, N-M178, N-B211, N-M2118, N-CST10760, N-Z1936, N-F4205, N-B202, N-B479, O-P186, O-M119, O-P31, O-M122, O-P201, O-M134, Q-M242, R1a-M198, R1a-PF6202, R1a-Y2395, R1a-CTS1211, R1a-Z92, R1a-Z93, R1b-M343, R1b-Y13887, R1b-M269, R1b-L51, R1b-Z2105, R2-M124, and T-M70. Genotyping was performed shortly after sample collection by real-time PCR with Open Array technology (Applied Biosystems, Foster City, CA, USA) using TaqMan assays (Applied Biosystems, Foster City, CA, USA), a 7900 HT (Applied Biosystems, Foster City, CA, USA) and a QuantStudio 12K Flex (Thermo Fisher Scientific, Waltham, MA, USA) amplification system. Branches of haplogroups Q-M242 and R1a-Z93 were identified by subgenotyping using TaqMan Open Array custom plates and a QuantStudio 12 Flex amplification system (Thermo Fisher Scientific, Waltham, MA, USA). There are 59 branches of different levels in haplogroup Q-M242 (M242, L275, Y1230, M378, Y5186, Y2990, Y2137, BZ3908, BZ321, YP745, YP1226, YP1095, Y2211, Y2200, M25, YP1669, L712, BZ1030, L713, YP1690, YP812, BZ1000, BZ664, YP4387, YP4385, Y18091, Y661, B143, B280, Z36048, M120, Y558, Y521, Y2659, L940, Y2677, SK1995, F1893, BZ1520, BZ1499, L53, YP4004, YP4000, BZ3140, YP4055, YP3952, L54, L804, M3, L330, YP1102, B31, Y12452, BZ99, Z35990, YP1691, YP771, L332, BZ427, BZ431) and 64 such branches in haplogroup R1a-Z93 (Z93, YP5585, Y52775, Y39884, YP1506, F17329, YP1522, FGC45895, KMS133, Y20793, Z94, Y40, M12253, YP294, Y3, Y27, F1417, Y148914, M605, Y9, Y7, Y945, Y30, Y29, Y944, Y28, Z2124, Z2122, Y43034, Y57, Y53, F2997, F2935, CTS6, Y2619, FGC18222, Y2630, Z2125, Z2123, SUR86, Y47, Y29452, BY30762, YP522, Y15121, Y874, Y151896, Y7094, Y5981, Y5983, YP454, YP6355, YP456, YP450, YP349, YP1558, YP5844, S23201, S21872, S15709, YP1456, YP1455, YP1543, YP413). These mutations form a hierarchical structure of branches within the studied haplogroup. The main male lineages of modern Tuva people studied carry the mutations Q-L330-YP1102 (TMRCA 4700 YBP), R1a-Z93-Y39884 (TMRCA 3600 YBP), R1a-Z93-YP1506 (TMRCA 3800 YBP), and R1a-Z93-Z94-Z2124-YP349-YP1456-YP1455-YP1543 (TMRCA 3400 YBP). TMRCA estimates are based on data from YFull [54].

The DNA concentration of all samples was adjusted to 35 ng/µL using a QIAGEN QIAgility (QIAGEN N.V., Venlo, The Netherlands) automated liquid dispensing station before genotyping. Y-SNP genotyping was performed with the inclusion of negative controls in each run. Alleles were determined by the fluorescence intensity of FAM and VIC dyes; a positive signal was considered to be at least 10% of the maximum observed in the same batch of samples. A sample was included in the analysis only if the derived state of at least two Y-SNP markers was unambiguously determined; samples that did not yield a result were discarded and are not included in the sample sizes. Since Y-SNPs were typed hierarchically, the correctness of each genotype was verified by internal phylogenetic consistency: the derived state of a downstream marker must be accompanied by the derived state of all upstream markers of the same lineage, and only mutually consistent combinations of markers were accepted as a reliable haplogroup determination. This requirement serves as a built-in positive control.

The position of Tuvans in the genetic space was estimated using the multidimensional scaling (MDS) method in the STATISTICA 9.0 program (StatSoft Inc., Tulsa, OK, USA) based on the frequencies of 16 main Y-haplogroups: C-M217(xM48,M407), C-M48, C-M407, D-M174, H-M69, I-M170, J-M304, N-M231(xP43,M178), N-P43, N-M178, O-M175, Q-M242, R1a-M198, R1b-M343, R2-M124, “others” (E, G, L, T, the sum of the frequencies of which in Tuvans is <0.2%). The analysis was performed for 25 populations from Southern Siberia and Central Asia (total sample *n* = 3677) using Nei’s pairwise genetic distance matrix (calculated in DJgenetics, [55]). The resulting configurations were visualized in Python 3.14 using the Matplotlib 3.11.0 library. Clusters in the MDS plot were identified visually.

**Figure 1:**
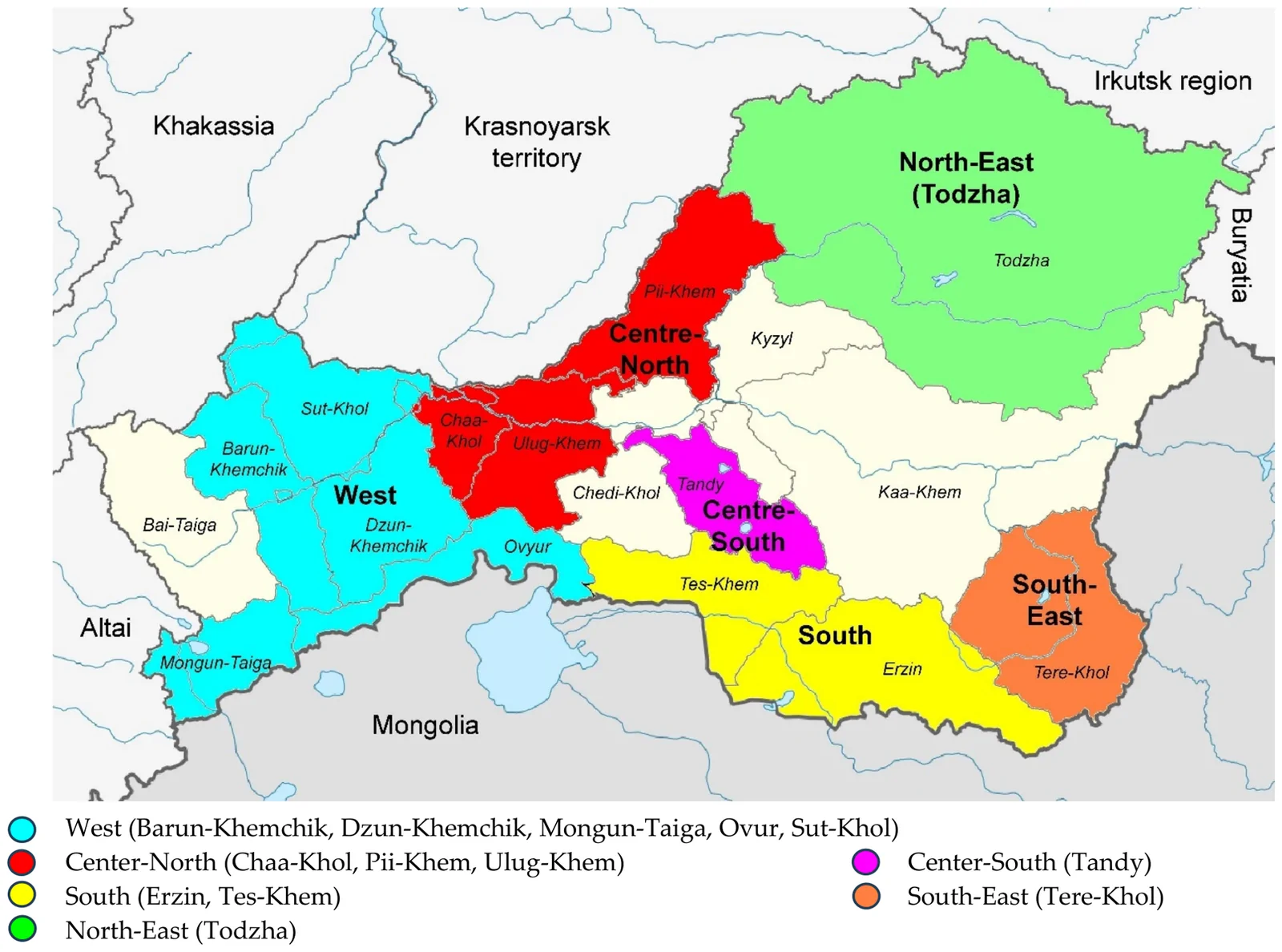
Map of the 6 regions of Tuva and their constituent districts where genetic samples were collected. Legend: Each region is indicated by a corresponding color.

The analysis of 633 samples from 31 Tuvan clans that live in almost all parts of Tuva (Supplementary Table S1, Figure 1) identified 221 carriers of the target haplogroups Q and R1a in 22 Tuvan clans, including Mongush (*n* = 34), Kyrgys (*n* = 31), Oyun (*n* = 15), Kol (*n* = 14), Choodu (*n* = 14), Ak (*n* = 13), Sat (*n* = 13), Khertek (*n* = 13), Baraan (*n*= 10), Soyan (*n* = 10), Dongak (*n*= 9), Irgit (*n* = 9), Oorzhak (*n* = 8), Ondar (*n* = 5), Todut (*n* = 4), Tulush (*n* = 4), Khovalyg (*n* = 4), Khoyuk (*n* = 4), Maady (*n* = 3), Kuular (*n* = 2), Salchak (*n* = 1), and Khomushku (*n* = 1).

A total of 202 samples representing haplogroups Q and R1a were successfully genotyped for the following 37 Y-STR markers using a Nanofor-05 genetic analyzer (Syntol LLC, Moscow, Russia): DYS389I, DYS389II, DYS390, DYS456, DYS19, DYS385a, DYS385b, DYS458,DYS437, DYS438, DYS448, GATA-H4, DYS391, DYS392, DYS393, DYS439, DYS635, DYS533, DYS504, DYS481, DYS525, DYS576, GATA-A10, DYS449, DYS445, DYS552, DYS537, DYS505, DYS460, DYF387S1a, DYF387S1b, DYS570, GGAAT1B07, DYS549, DYS643, DYS627, DYS518 (Supplementary Table S2). The remaining 19 samples were not properly genotyped due to DNA degradation.

Phylogenetic networks for Y-STR haplotypes were constructed using the median-joining algorithm [56] implemented in Network v.10.2.0.0. Network images were created in Network Publisher v.2.1.2.5. The weight of each of 37 STR markers was assumed to be 10 at ε = 0. Time to the most recent common ancestor (TMRCA) was estimated using the ASD method [57]. The average mutation rate was assumed to be 0.0045 per locus per generation for 27 Y-STR haplotypes (ancient samples) and 0.0039 per locus per generation for 37-Y-STR haplotypes (modern samples) [58,59]. The average generation time was assumed to be 31.5 years [60]. The Y-STR markers handling for estimating TMRCA and the method for calculating confidence intervals are described in [61].

For modern and ancient Y-STR haplotypes for which no information regarding Y-SNP branches was available, predictions were made by comparing the Y-STR marker values of the analyzed sample with those of similar samples for which the corresponding Y-SNP branch was known. To validate the predictions, a comparative analysis was conducted using modal haplotypes from the YFull global Y-chromosome tree [54].

To analyze the structure of the ancient Y gene pool, we used published data [42,46,47,48,49,50,51,52], which we arranged by historical and cultural periods (Table 1). For ancient samples, Y-chromosome branches reported in the literature were compared with the data in the YFull database [54], haplotree.info (https://docs.google.com/spreadsheets/d/1xfeK8HvVjkCY7mKj3WEKAjapAqltooWJMptY0nStKbo/edit?gid=1942507897#gid=1942507897, accessed on 26 December 2025), FamilyTreeDNA Discover™ (https://discover.familytreedna.com/, accessed on 13 January 2026), TheYtree (https://www.theytree.com/tree/, accessed on 13 January 2026), and the summary table from [42].

Our modern samples were genotyped for 37 Y-STR markers, of which 27 were from the Yfiler Plus kit used to analyze the ancient samples from the Tuvan Scytho-Siberian period (*n* = 45, Table 1) [47,48,49,50]. This allowed us to construct phylogenetic networks that integrated both ancient and modern datasets. Importantly, there was a gap of at least 2300 years (~73 generations) between the modern and ancient donors. If the ancient donor had been a direct ancestor of modern carriers of the analyzed Y-STR haplotype, then we would expect an average of 0.0045 × 73 = 0.33 Y-STR mutations per locus to have occurred within this time frame. If all such mutations had been single-step, then an average of 0.33 × 27 = 8.9 single-step mutations would have occurred so far in one 27-marker haplotype. However, the estimated number of single-step mutations does not coincide with their actual number because of reversion, in which a mutated Y-STR allele mutates back to its previous state. The symmetric stepwise mutation model predicts an average number of observed mutations at 7.6 per haplotype (see Appendix A). Thus, in phylogenetic networks, haplotypes of modern descendants should cluster around the points of their most recent common ancestor, with an average number of 7–8 observed steps. If, however, the centers of the ancient and modern clusters do not coincide, then these clusters had different most recent common ancestors.

## 3. Results

### 3.1. The Position of the Tuvan Gene Pool Among the Adjacent Populations

**Figure 2:**
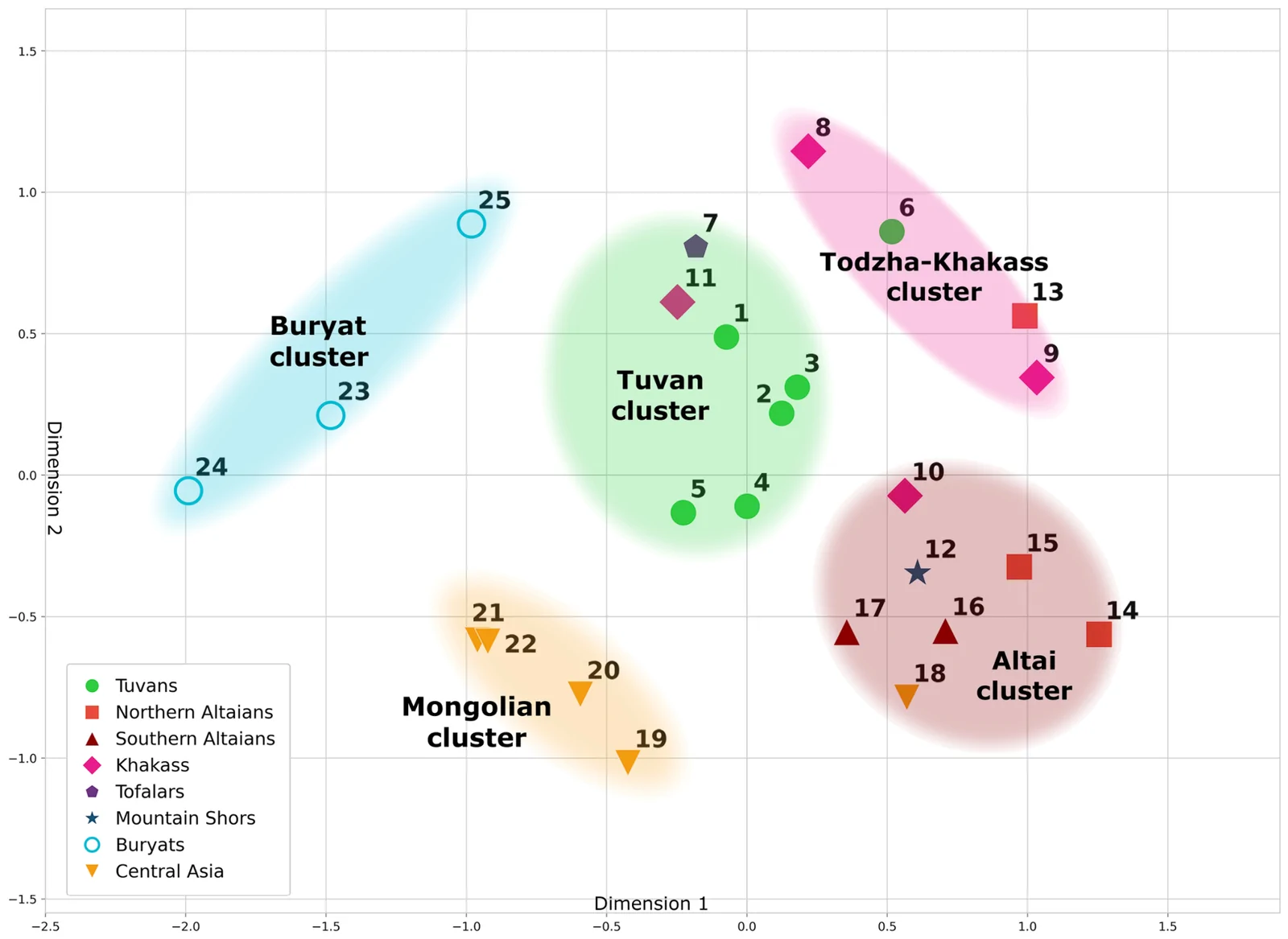
Multidimensional scaling plot of South Siberian and Central Asian populations based on 16 major haplogroups; stress 0.16, alienation 0.18. Legend symbols: 1—Tuvans: West (*n* = 142); 2—Tuvans: Center-North (*n* = 104); 3—Tuvans: Center-South (*n* = 87); 4—Tuvans: South (*n* = 120); 5—Tuvans: South-East (*n* = 90); 6—Tuvans: North-East (*n* = 90); 7—Tofalars (*n* = 50); 8—Khakass: Kachins (*n* = 76); 9—Khakass: Koibals (*n* = 24); 10—Khakass: Sagay-Beltirs (*n* = 24); 11—Khakass: Sagay-Biryusa (*n* = 38); 12—Mountain Shors (*n* = 108); 13—Northern Altaians: Chelkans (*n* = 65); 14—Northern Altaians: Kumandins (*n* = 56); 15—Northern Altaians: Tubalar (*n* = 83); 16—Southern Altaians: Altai-Kizhi (*n* = 74); 17—Southern Altaians: Telengits (*n* = 129); 18—Kyrgyz (*n* = 294); 19—Kazakhs (*n* = 366); 20—Mongolians: West (*n* = 387); 21—Mongolians: Center (*n* = 310); 22—Mongolians: East (*n* = 312); 23—Buryats of Buryatia (*n* = 267); 24—Buryats of the Irkutsk Region (*n* = 121); 25—Buryats of Transbaikalia (*n* = 228). Total sample size: 3645.

Tuvans live at the junction of South Siberia and Central Asia, so their gene pool could have been affected by the interactions with the adjacent populations. On the multidimensional scaling plot for the entire list of haplogroups (Figure 2), nearly all Tuvan populations cluster with the neighboring Tofalar population of the Irkutsk Region and the Sagais, who are the largest subgroup of the Khakass. The Todzha Tuvans are the only Tuvan population that falls under a different cluster together with the Khakass (Koibals and Kachins) and the Altaian Chelkans. Other Altaians form a separate cluster with the Shor and the Kyrgyz. The Buryat constitute a separate cluster. All Mongol populations make up the Mongol cluster, which also includes the Kazakh.

**Figure 3:**
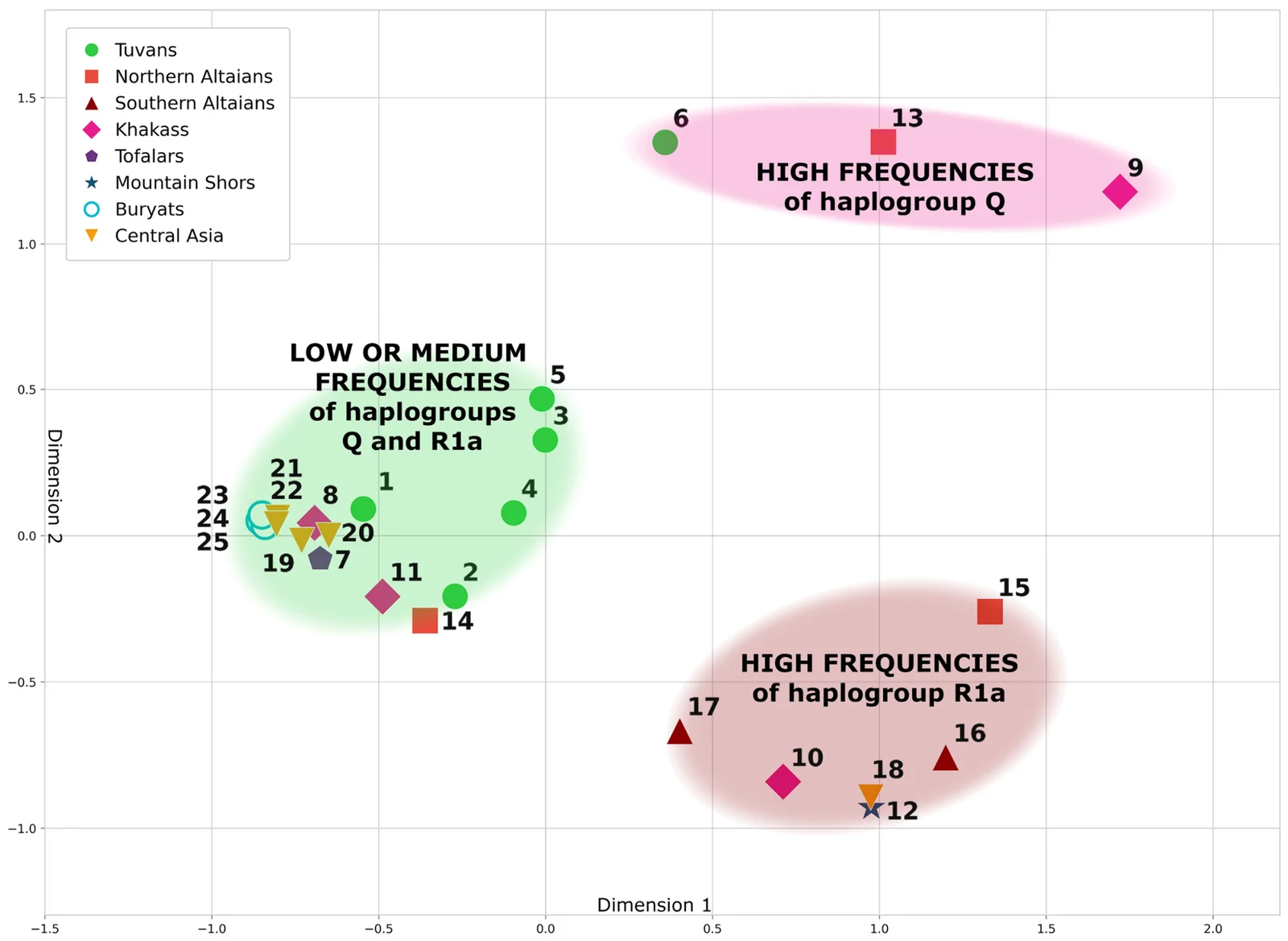
Multidimensional scaling plot of South Siberian and Central Asian populations based on haplogroups R1a and Q only; stress 0.003, alienation 0.005. Legend symbols: 1—Tuvans: West (*n* = 142, Q = 12, R1a = 14); 2—Tuvans: Center-North (*n* = 104, Q = 23, R1a = 19); 3—Tuvans: Center-South (*n* = 87, Q = 4, R1a = 20); 4—Tuvans: South (*n* = 120, Q = 18, R1a = 25); 5—Tuvans: South-East (*n* = 90, Q = 23, R1a = 14); 6—Tuvans: North-East (*n* = 90, Q = 46, R1a = 3); 7—Tofalars (*n* = 50, Q = 0, R1a = 5); 8—Khakass: Kachins (*n* = 76, Q = 3, R1a = 5); 9—Khakass: Koibals (*n* = 24, Q = 14, R1a = 7); 10—Khakass: Sagay-Beltirs (*n* = 24, Q = 0, R1a = 13); 11—Khakass: Sagay-Biryusa (*n* = 38, Q = 0, R1a = 7); 12—Mountain Shors (*n* = 108, Q = 0, R1a = 66); 13—Northern Altaians: Chelkans (*n* = 65, Q = 36, R1a = 10); 14—Northern Altaians: Kumandins (*n* = 56, Q = 0, R1a = 13); 15—Northern Altaians: Tubalar (*n* = 83, Q = 18, R1a = 41); 16—Southern Altaians: Altai-Kizhi (*n* = 74, Q = 6, R1a = 44); 17—Southern Altaians: Telengits (*n* = 129, Q = 2, R1a = 58); 18—Kyrgyz (*n* = 294, Q = 3, R1a = 176); 19—Kazakhs (*n* = 366, Q = 4, R1a = 26); 20—Mongolians: West (*n* = 387, Q = 14, R1a = 34); 21—Mongolians: Center (*n* = 310, Q = 5, R1a = 7); 22—Mongolians: East (*n* = 312, Q = 3, R1a = 8); 23—Buryats of Buryatia (*n* = 267, Q = 0, R1a = 0); 24—Buryats of the Irkutsk Region (*n* = 121, Q = 1, R1a = 0); 25—Buryats of Transbaikalia (*n* = 228, Q = 0, R1a = 2). Total sample size: 3645 including 235 carriers of Q-M242 and 617 carriers of R1a-M198.

The multidimensional scaling plot constructed for haplogroups Q and R1a (Figure 3) provides some insights into their role in the genetic interaction between the populations. The plot shows that the populations form three visually distinguishable clusters. In this analysis, the impact of Central Asian haplogroups C and O is ignored; this brings Central Asian and Buryat populations much closer to the massive Tuvan cluster, where Q and R1a frequencies are at the regional average. The Todzha-Khakass cluster is distinguished by the increased frequency of haplogroup Q (51% in Todzha Tuvans, 55% in Chelkans, 58% in Koibals). The Altaian cluster is characterized by the increased frequency of R1a (49% in Tubalars, 59% in Altai-Kizhi, 60% in Kyrgyz, 61% in Shors). The Telengit, Sagai and Kumandin populations form a “bridge” connecting the Altaian cluster to the main body of gene pools with medium R1a and Q frequencies. The direct Q-versus-R1a frequency plot demonstrates the same visually apparent groupings (see Supplementary Figure S1). No additional cluster analysis is essential.

In the Tuvans, haplogroups Q and R1a collectively constitute about one-third of the gene pool, occurring at 20% and 15% on average, respectively (Table 2). However, their frequencies vary substantially across different parts of Tuva (Table 2, Figure 3). The frequencies of haplogroup Q range from 5% in the Center-South region to 51% in the North-East in Todzha Tuvans. R1a frequencies follow the opposite pattern: they range from 23% in the Center-South region of Tuva to 3% in the North-East in Todzha Tuvans. Between the clans, the frequencies of major Y-chromosome branches are even more heterogeneous (Table 2; Supplementary Table S3). For example, the frequency of R1a estimated for 13 clans with a sample size of *n* ≥ 20 varies from 4% in the Ak clan to 42% in the Oyun clan. Moreover, there is substantial variation even within one clan. For example, haplogroup C-M407 occurs at only 7% in the representatives of the Kyrgys clan in the South region and reaches 30% in the South-East. This variation between the genetic portraits of the clans plays a significant role in the structure of the Tuvan gene pool. Therefore, the phylogeny of Q and R1a is analyzed below in two ways: collectively for all Tuvans and in the context of their clan affiliation.

Ancient DNA discovered across Tuva demonstrates an important phenomenon: during Tuvan ethnogenesis, the total frequency of Q and R1a dropped from 85% (35/41; 95% CI 71–94%) in Scytho-Siberian populations to 35% (221/633; 95% CI 31–39%) in modern Tuvans. The ancient specimens found in Tuva (Table 1, Supplementary Table S4) represent the Pre-Scythian times (3300–2900 YBP, the Mongun-Taiga culture below referred to as MT), and the Scytho-Siberian period (2800–2200 YBP, the Aldy-Bel and the Uyuk-Sagly cultures below referred to as AB and US, respectively). Ancient DNA samples from Tuva are characterized by very low Y-chromosome diversity (Supplementary Table S4): only six haplogroups were identified by our prediction (Table 1), including three major haplogroups (46% R1a-M513; 36% Q-L330; 9% N-Y147969) and three minor ones (5% C-M217; 2% I; 2% R1b).

### 3.2. Phylogenetic Analysis of Haplogroup Q-M242 in Ancient and Modern Population of South Siberia

**Figure 4:**
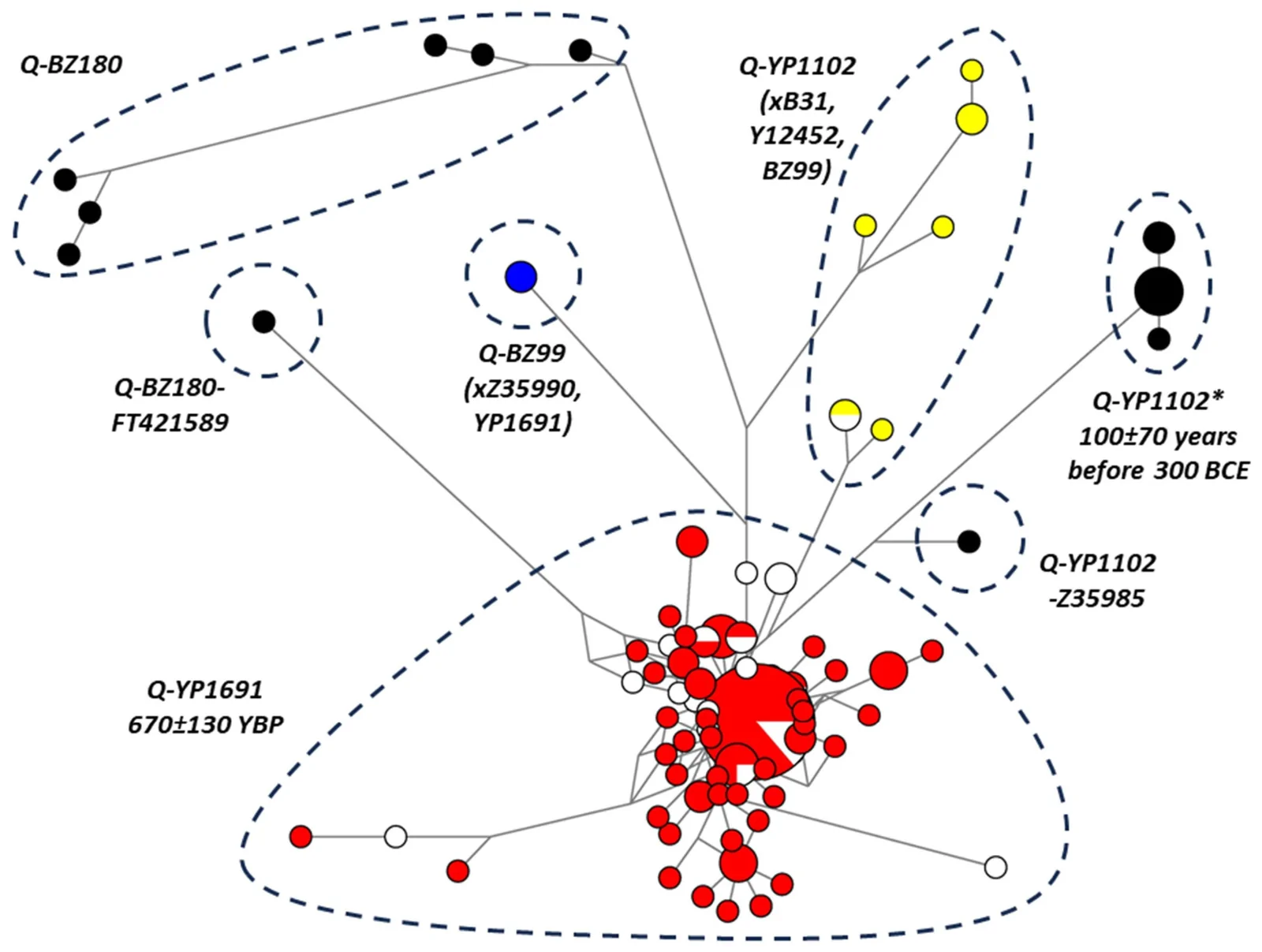
The phylogenetic network of modern and ancient samples based on 27 Y-STR marker haplotypes of haplogroup Q-L330, *n* = 133. Y-STR haplotypes are represented by circles with area proportional to the number of individuals with that haplotype (from 1 to 29). Branch lengths are proportional to the number of one-repeat mutations separating the two haplotypes. The dashed borders of modern samples highlight clusters of directly genotyped Y-SNP color-coded branches. Modern samples without genotyping are shown in white. Haplogroups Q-YP1691, Q-BZ99(xZ35990,YP1691), Q-YP1102(xB31,Y12452,BZ99) are color-coded by red, blue, and yellow, respectively. Nodes with both directly genotyped samples and samples without genotyping are indicated by pie chart divisions. Ancient samples are color-coded by black. The dashed borders of ancient samples indicate visually inferred clusters confirmed by the Y-SNP branch prediction based on Y-STR haplotypes. For some clusters, the time to the most recent common ancestor is given.

The phylogenetic network for haplogroup Q-M242 built from 117 modern and 16 ancient 27 Y-STR marker haplotypes shows that all 133 Y-STR haplotypes are located on the branches of subhaplogroup Q-L330 (Figure 4). This Siberian subhaplogroup emerged at~ 15,500 YBP and split into two branches (YP1102 and YP771) at 8000 YBP [54]. The most ancient subhaplogroup Q-L330 sample (7800 YBP) was found in the vicinity of Krasnoyarsk (Supplementary Table S5) [42].

The phylogenetic network (Figure 4) demonstrates that the ancient and modern Y-STR haplotypes from Tuva form several distinct clusters that differ sharply by the number of constituent haplotypes.

All modern Y-STR haplotypes for which the Y-SNP branch has not been determined are indicated by white circles in Figure 4. These haplotypes are interspersed in a large array of the tested modern haplotypes which form Q-YP1691 and Q-YP1102(xB31,Y12452,BZ99) clusters, so the prediction of their branches is strongly supported by Y-STR similarity. Y-STR haplotypes of ancient samples are located at a significant distance from the clusters of modern samples. Thirteen tandem repeats at the DYS437 locus indicate the haplogroup Q-L330 (Supplementary Table S4). Additionally, three ancient samples SU03, SU04, and SU05 were genotyped as Q-L330 in the study [50]. We predicted more detailed branches for ancient samples by comparing Y-STR alleles with the YFull modal haplotypes for the Q-L330 branches [54].

Sixteen ancient samples representing subhaplogroup Q-L330 (Supplementary Table S4) are distributed across four of its branches (Figure 4). Seven samples represent two subbranches of BZ180, which are absent in our sample set of modern Tuvans. Of them, one sample from the Scythian period (AB01) belongs to subbranch Q-BZ180-FT421589. Six samples belong to subbranch Q-YP771-BZ180; of them, one is from the Pre-Scythian times (MT01) and the remaining five are from the archeological cultures of the Scythian period (AB02, US01-US04). Another nine ancient samples (US05-US13) are associated with the Uyuk-Sagly period and represent subhaplogroup Q-YP1102: one belongs to subbranch Q-YP1102-Z35985, and Y-STRs of the remaining eight constitute a very young (TMRCA ≤ 300 years before the time these men were alive) compact Q-YP1102* cluster.

The majority of the analyzed modern Tuvan Q-L330 samples (*n* = 117) form a large cluster (*n* = 107) with a pronounced founder effect on subbranch Q-YP1691 (Figure 4). The rest (*n* = 10) are distributed between two clusters: Q-YP1102(xB31,Y12452,BZ99) (*n* = 8) and Q-BZ99(xZ35990,YP1691) (*n* = 2).

In our dataset, 91% of the samples representing modern Tuvan carriers of macrohaplogroup Q-M242 belong to Q-YP1691. According to YFull estimates for Y-SNP mutations, this branch emerged at ~2800 YBP and split at ~2200 YBP [54]. This estimate agrees with the archeological dating for the Scytho-Siberian period in Tuva (9th–3rd centuries BCE). The accumulated evidence suggests that at that time there should have been few carriers of the YP1691 mutation: they were not found in Tuva but existed in the Medieval Ust-Ishim culture within the territory of today’s Omsk region (~950 YBP [62], Supplementary Table S5). This hypothesis does not contradict our age estimate for the Q-YP1691 haplotypes cluster of modern Tuvans, which dates MRCA of the sampled modern Y-STR cluster to a later period (670 ± 130 YBP, where 130 years represent one standard error of the mean). At the same time, a young TMRCA branch does not necessarily indicate migration; the ancestors of this branch could have lived in the same area.

It can be considered reliably established that the Scytho-Siberian Q-YP771-BZ180 lineages has not been preserved in significant numbers among modern Tuvans. However, continuity of Q lineages in Tuva is observed at the level of the fairly old haplogroup Q-YP1102 (the YFull’s TMRCA ~4700 YBP [54]), which forms a bridge between ancient and modern samples. Q-YP1102 dominates the gene pools of Bronze Age Yeniseian populations [33,41,42] and occurs in 20% of modern Tuvans as branches Q-YP1691, Q-BZ99(xZ35990,YP1691), and Q-YP1102(xB31,Y12452,BZ99). However, the ancient cluster of Y-STR haplotypes Q-YP1102*, as well as the haplotype Q-Z35985 of the US05 sample, do not coincide with the clusters of modern Tuvans (Supplementary Tables S2 and S4). The ancient Yeniseian substrate persists in the modern Tuvans mainly in the form of Q-YP1691 lineages.

**Figure 5:**
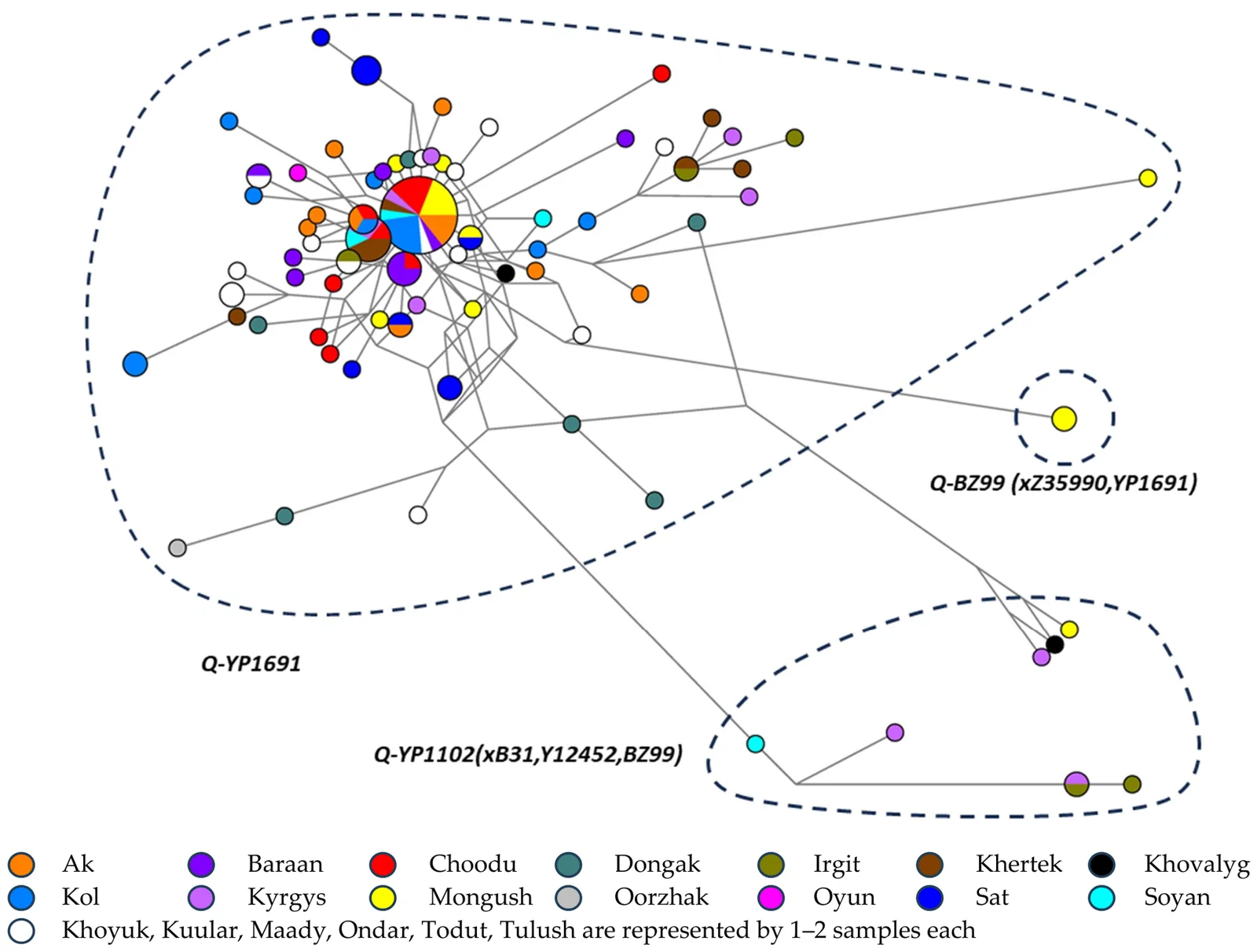
The phylogenetic network of modern Tuvan samples based on 37 Y-STR marker haplotypes of haplogroup Q-YP1102, *n* = 117. Y-STR haplotypes are represented by circles with areas proportional to the number of individuals with that haplotype (from 1 to 21). Branch lengths are proportional to the number of one-repeat mutations separating the two haplotypes. The dashed borders highlight clusters of directly genotyped Y-SNP branches. Haplotypes are color-coded by Tuvan clans (see key), with haplotype sharing indicated by pie chart divisions.

The phylogenetic network (Figure 5), based on 117 modern 37-marker haplotypes of Tuvans illustrates the distribution of haplotypes across Tuvan clans. The network shows the same three branches of subhaplogroup Q-YP1102. Branch Q-BZ99(Z35990,YP1691) is represented by 2 samples of the Mongush clan from Central Tuva. Branch Q-YP1102(xB31,Y12452,BZ99) is represented by the samples from the South-East, South, and the Center regions of Tuva: Kyrgys (*n* = 3), Irgit (*n* = 2), Khovalyg (*n* = 1), Mongush (*n* = 1), and Soyan (*n* = 1). The large Q-YP1691 cluster accumulates samples (47 out of 107) that represent the North-East clans from the Todzha district: Kol (*n* = 13), Ak (*n* = 11), Baraan (*n* = 9), and Choodu (*n* = 5). South-East and South regions are represented by 31 samples, mostly by the Choodu (*n* = 6), Khertek (*n* = 7) and Kyrgys (*n* = 7) clans. This confirms a previously reported [33] gradient of Q-YP1691 frequency that increases from west to east and reaches its peak in Todzha Tuvans.

Subhaplogroup Q-YP1691 prevails in Kets and Selkups [63] and is widespread in the modern indigenous populations of South and West Siberia [our own data], specifically in Southern Altaians (Altai-Kizhi and Telengit), Koibal Khakass, Siberian Tatars (Yalutorovsk, Iskero-Tobolsk, Barabinsk, Tumen), Khants, and in the populations of Mongolia (Uriankhai from Bayan-Ulgii and Khovd aimags). We also assigned three published Kyrgyz samples of 23-marker Y-STR haplotypes to this subhaplogroup [64] (Supplementary Table S6). Branch Q-BZ99(xZ35990, YP1691) is rarely observed in Tuvans (2%), but we found it in many populations of Siberia and Central Asia [our own data]: Koibal Khakass, Iskero-Siberian Tatars, Khants, and Uzbeks from the Khorazm region of Uzbekistan. We also identified distinct, previously unidentified branches of haplogroup Q-YP1102 in the Tuvans, Kachin Khakass, Siberian Barabinsk Tatars, Mongols, Kalmyks, and Uzbeks from the Khorazm region of Uzbekistan [our own data]. Branch Q-YP1102-Y12452 occurs in the South of Kazakhstan [our own data, [65,66,67]] (Supplementary Table S6). Summing up, haplogroup Q-YP1102 marks Yeniseian descent. Today, its frequency reaches 20% in Tuvans (Table 2), 16% in Khakass, 14% in Khants, 6% in Southern Altaians [our own data], 26% in Chulyms [68], and peaks in Kets at 86–91% and Selkups at 58–66% [69,70] (Supplementary Table S6). In Central Asia, Q-YP1102 has lower frequencies: 2–3% in Southern Kazakhs and Uzbeks, 1–2% in Mongols and Kalmyks [our own data], and below 1% in the Kyrgyz of Kyrgyzstan [64].

### 3.3. Phylogenetic Analysis of Haplogroup R1a-M513 in Ancient and Modern Population of South Siberia

One of the ancient samples (MT02, Supplementary Table S4) was excluded from the analysis of R1a phylogeny because its association with haplogroup R1a [48] has not be correct: comparison of its Y-STR alleles with modal YFull haplotypes provisionally placed the MT02 sample on the R1b-PH155-PH200-Y104457 branch [54]. Further, two Y-STR haplotypes of modern Tuvans, directly genotyped as R1b-M343(xY13887,M269), appear belong to the same lineage as the MT02 (Supplementary Table S6). Subhaplogroup R1b-PH200 was widespread in the second half of the 1st millennium BCE in what is now Chinese Xinjiang and in the Khovd aimag in Mongolia [71,72,73,74] (Supplementary Table S5); it also occurs in modern Xinjiang Uyghurs [75,76] (Supplementary Table S6).

**Figure 6:**
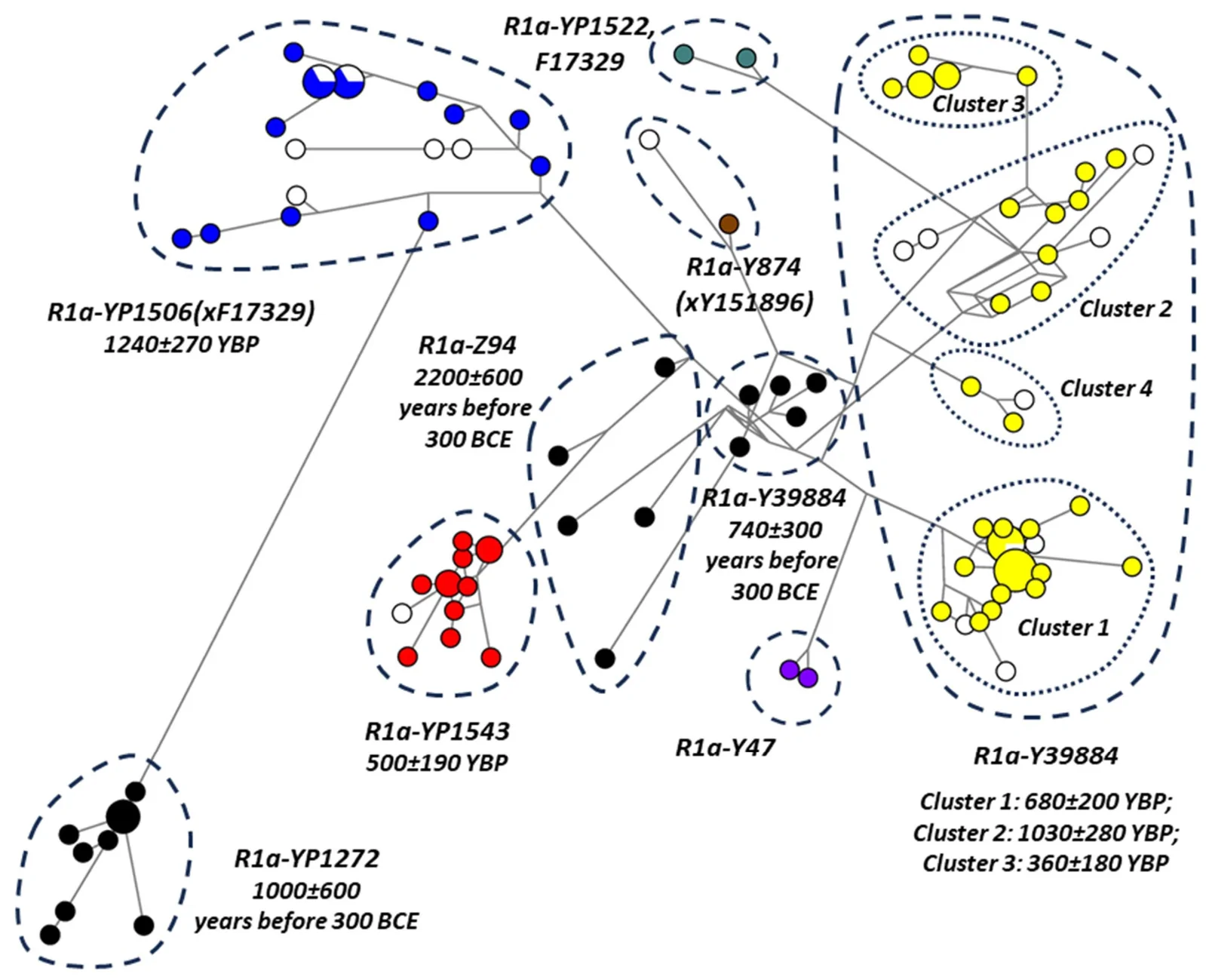
The phylogenetic network of modern and ancient samples based on 27 Y-STR marker haplotypes of haplogroup R1a-M513, *n* = 105. Y-STR haplotypes are represented by circles with areas proportional to the number of individuals with that haplotype (from 1 to 5). Branch lengths are proportional to the number of one-repeat mutations separating the two haplotypes. The dashed borders of modern samples highlight clusters of directly genotyped Y-SNP color-coded branches. The dotted borders highlight visually inferred clusters within haplogroup R1a-Y39884 (their Y-SNPs are unknown). Modern samples without genotyping are shown in white. Haplogroups R1a-YP1543, R1a-YP1506(xF17329), R1a-Y39884, R1a-YP1522,F17329, R1a-Y874(xY151896), and R1a-Y47 are color-coded by red, blue, yellow, dark blue-green, dark brown, and dark saturated purple, respectively. Nodes with both directly genotyped samples and samples without genotyping are indicated by pie chart divisions. Ancient samples are color-coded by black. The dashed borders of the ancient clusters are visually grouped around the directly genotyped samples, namely three ancient samples in R1a-Z94, two ancient samples in R1a-Y39884, and one ancient sample in R1a-YP1272, respectively. For some clusters, the time to the most recent common ancestor is given.

After excluding sample MT02, a phylogenetic network for haplogroup R1a-M513 was constructed using 105 samples: 20 ancient samples of 27-marker Y-STR haplotypes and 85 modern samples from the Tuvan population. Clusters of modern and ancient Y-STR haplotypes R1a-M513 (Figure 6) do not coincide with each other, as is the case with haplogroup Q-M242 (Figure 4). Modern white-color haplotypes without Y-SNP subtyping are interspersed within clusters with tested mutations.

In the phylogenetic network (Figure 6), there is a cluster formed by the samples that represent the relict macrohaplogroup R1a-M513(xM417) characterized by 14 tandem repeats in the DYS392 locus. This cluster includes 10 ancient samples of the Scytho-Siberian Tuvan population (AB03-AB10, US14, US15). Their haplotypes can be assigned with high probability to haplogroup R1a-YP1272, since the US15 sample, which was directly genotyped for F3054 mutation, belongs to this haplogroup [50]. R1a-YP1272 lineages of the Scytho-Siberian period in Tuva have not been observed in our sample set. A product of sequential mutations M513-M459-M735-YP1306-YP1272, R1a-YP1272, is extremely rare in the modern Eurasian population [77]. So far, the ancient samples of its parent haplogroup R1a-YP1306 (archeological dating ~13,000–4000 YBP) have been found only in Europe (Supplementary Table S5): in the Arkhangelsk region of Russia (~12,700 YBP [78]), the Republic of Karelia (~8450 YBP [79]), Estonia (~5500 YBP [80]), and in the Voronezh region of Russia (~4000 YBP [81]).

The remaining ancient and modern samples in the phylogenetic network (Figure 6) belong to the branches of R1a-M198-Z93, a haplogroup widespread in the steppe zone of Eurasia. Previously, ancient DNA samples US16–US20 were genotyped for Y-SNP mutations Z93 and Z94 [50]; this helped us to identify two branches of R1a-Z93: R1a-Z94 and R1a-FGC82884-Y39884 (Supplementary Table S4). All samples of the Scytho-Siberian period with haplotypes R1a-Z93 are closely arranged in the center of the network (Figure 6): the distance between the ancient haplotypes is quite short, because only 2000 years elapsed since R1a-Z93 split into R1a-FGC82884 and R1a-Z94 [54].

Five ancient samples from Tuva (MT03, AB11, US16–US18) belong to R1a-Z94 branches (Figure 6). Sample CGG_2_021496 from the Aimyrlyg necropolis in Tuva also represents R1a-Z94 [51]. This haplogroup was very common in the Scytho-Siberian world. Its branch R1a-Z94-S23592-YP1456 occurred in Tuva (I0577) [46] and the Tasmola culture of Kazakhstan (KKM001, KYZ001, TAL003, TAL004, CSP003, CSP004) [82]. In Mongolia (2300–2200 YBP, Chandman [32,72]), haplogroup R1a is represented mainly by its branch R1a-Z94-S23592 (CHN008, CHN012, I6224, I6233, I7030), including by two samples of branch R1a-S23592-YP1456 (CHN001, I7027) [32,72] (Supplementary Table S5).

Five other specimens from the Scytho-Siberian period in Tuva (AB12, AB13, US19–US21) form a compact cluster R1a-Y39884 that emerged ~700 years before those individuals lived (Figure 6). Unlike R1a-Z94, haplogroup R1a-Y39884 was rare during the Scytho-Siberian period: it was previously discovered in Mongolia (I7024, *n* = 1 [72]) and at the foothills of the Tian Shan mountains in Kazakhstan in a sample representing the ancient Wusun population (DA223, *n* = 1 [83]). Neither Western nor Eastern Scythians from the territories of modern Russia had this haplogroup: it was first discovered in five samples in this study (AB12, AB13, US19–US21, Supplementary Table S4). In later periods, R1a-Y39884 occurred in the carriers of the Tashtyk culture of Khakassia (KE9609, *n* = 1, 1700 YBP [84]). Another specimen from the Tashtyk culture (S34 [40]) has 17 Y-STR marker matches with the modal haplotype of the Scytho-Siberian samples from Tuva in the R1a-Y39884 cluster (US20, Supplementary Table S4). The carriers of the Scytho-Siberian Tagar culture with DYS390 = 24 and DYS19 = 17 (S24, S32 [40]) can be provisionally assigned to this branch too.

**Figure 7:**
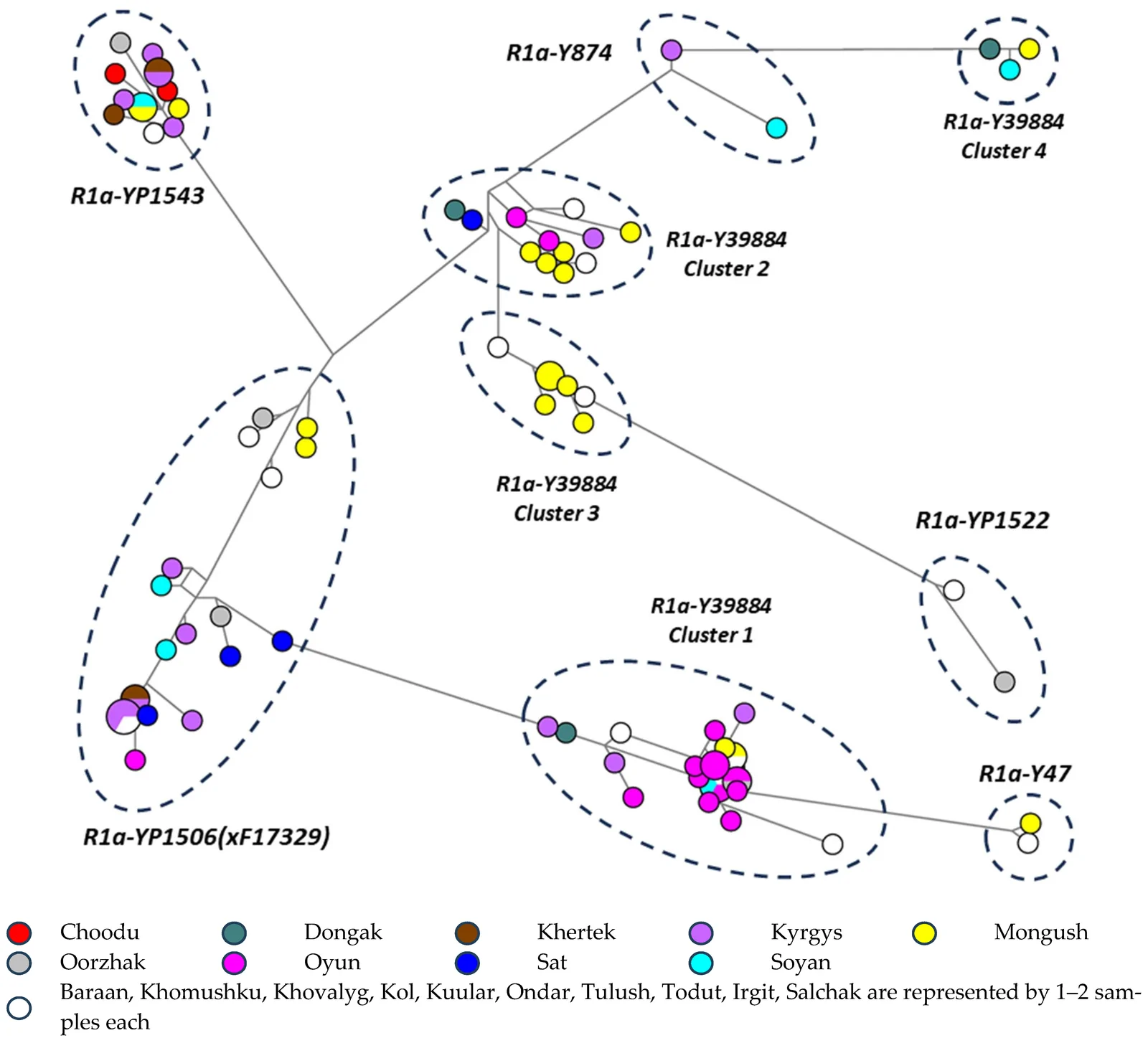
The phylogenetic network of modern Tuvan samples based on 37 Y-STR marker haplotypes of haplogroup R1a-Z93, *n* = 85. Y-STR haplotypes are represented by circles with areas proportional to the number of individuals with that haplotype (from 1 to 3). Branch lengths are proportional to the number of one-repeat mutations separating the two haplotypes. The dashed borders highlight clusters of directly genotyped Y-SNP branches. Haplotypes are color-coded by Tuvan clans (see key), with haplotype sharing indicated by pie chart divisions.

The phylogenetic network for haplogroup R1a-Z93 based on 37-marker haplotypes of 85 modern Tuvan samples is more detailed (Figure 7). It shows nine clusters that are distinct from one another due to accumulated Y-STR mutations. Modern Tuvan carriers of R1a-Z94 constitute three small clusters corresponding to branches YP1543, Y874(xY151896), and Y47. The larger (12%) haplogroup R1a-FGC82884 is divided into Y39884, YP1506(xF17329), and YP1522/F17329 clusters. R1a-Y39884 (Figure 7) is somewhat clan specific: half of the samples in cluster 1 are from the Oyun clan, and half of the samples in clusters 2–4 are from the Mongush clan. The main constituents of the R1a-YP1506(xF17329) cluster are the Kyrgys, Mongush, Oorzhak, Sat, and Soyan clans. The greatest contribution to the R1a-YP1543 cluster is made by the Kyrgys, Mongush, Choodu, and Khertek clans. The dominance of individual clans is reflected in the geographic differentiation of the main clusters: all four clusters of R1a-Y39884 concentrate in Central Tuva; all samples from cluster 1 are from the Center-South region of Tuva (the Tandy region). Haplotypes of R1a-YP1506(xF17329) and R1a-YP1543 occur in the South and South-East regions (Figure 7).

The accumulated body of evidence on the ancient and modern R1a-Y39884 samples suggests that this haplogroup emerged in Siberia at ~3800–3600 YBP [54] and has a strong connection with the Andronovo culture. Outside Tuva, samples of haplogroup R1a-Y39884 are found in the modern population of South Siberia (Khakass, Shor, Northern Altaian [our own data]) and less often in Central Asia in Mongol, Kazakh and Kyrgyz populations [our own data]. This leads to a hypothesis that the ancestors of modern R1a-Y39884 carriers could have lived in Tuva during the Scythian period.

Haplogroup R1a-YP1506 occurs (Supplementary Table S6) in South Siberia (in the Khakass, Shor and Altaian populations [our own data]), Central Asia (in the Pamir Kyrgyz [our own data], South Kyrgyzstan [64] and the Xinjiang Kyrgyz of China [85]). Its daughter branch R1a-YP1522 is common in Southern Altaians, reaching the frequency of 38% in Telengits and 23% in Altai Kizhis [our own data].

Our findings on haplogroup R1a-Z93 variants in Tuvans are consistent with the hypothesis [37] that the diversity of branches and the absence of a pronounced founder effect indicate the ancientness of R1a-Z93 lineages in Tuvans and the possibility of long-term migration of R1a-Z93 branches into the ancestral gene pool of the Tuvans. On the other hand, it should be taken into account that the age of Y-STR clusters varies from 1200 to 400 YBP (Figure 6), that is, their most recent common ancestor lived in the Middle Ages or even in the New Age.

Haplogroup R1a-Z94-YP1543 (2–3%), rarely found in Tuvans, occurs in about one-third of the Kyrgyz of Kyrgyzstan (34%) and the Altai-Kizhi (32%). A few samples belong to other branches of R1a-Z94 (R1a-Z2123-Y874(xY151896) and R1a-Z2123-Y47), which are widespread in South and West Eurasia, but always at low frequencies.

### 3.4. Distribution of Haplogroup N-M231

The big picture of Tuvan ethnogenesis will not be complete without the relict haplogroup N-Y147969 detected in the ancient samples from Tuva (*n* = 4, Supplementary Table S4). This haplogroup was widespread in ancient times but most of its branches are now extinct [86]. Although it is not found in modern Tuvans, its modern carriers closest to Tuva are found among the Northern Altaians (Kumandins, Tubalars and Chelkans) at 12% frequency [our own data]).

The ethnic and cultural composition of the haplogroups represented by the published ancient samples [46,47,48,49,50,52] does not suggest that haplogroups N-P43 and N-M178 found in modern Tuvans were inherited from the Scytho-Siberian population of the Eerbek River valley. However, haplogroup N-P43 occurred in the synchronous Pazyryk culture of Mountainous Altai (2600–2300 YBP [87]). The arrival of haplogroup N-M178-F4205 and C-M217 branches in Tuva might have been related to the political and economic activity of nomadic Asian empires, like Xiongnu, the Turkic and Uyghur Khaganates, Mongol, etc. [88].

Most of the ancient Tuvan DNA comes from the Eerbek River valley. The Eerbek basin (~490 sq. km.) makes up only 0.3% of Tuva’s territory (~168,600 sq. km). So, to solve the problem of genetic continuity of N-M231 branches, ancient specimens are needed from other parts of Tuva followed by their genetic data of high resolution.

## 4. Discussion

The revealed structure of the Tuvan gene pool and the accumulated knowledge about the ancient DNA of the Tuvans, who are the largest ethnic group in South Siberia, shed light on some aspects of South Siberian ethnogenesis in the context of Q and R1a phylogeography. It should be noted that Y-chromosome variation reflects only paternal ancestry and therefore represents a single component of population history.

The frequency of Y-chromosome haplogroups in modern Tuvans has been reliably studied due to a large sample (*n* = 633) and coverage of all regions of Tuva. Another study obtained frequencies consistent with our data (Table 2) [29]. The sample set of ancient Y-STR haplotypes from Tuva is significantly smaller (*n* = 45) and represents the ancient population of the Eerbek River valley located on the border of Southern Siberia and Mongolia. Limited ancient DNA data make the authors cautious in drawing conclusions.

In the Pre-Scythian period (3400–2900 YBP, Late Bronze Age), burials practiced in Tuva and Mongolia were of the Mongun-Taiga type (MT), including khirigsuur mounds in Tuva and synchronous Sagsai burials in the North and West Mongolia. They did not contain any grave goods, which distinguishes them from Scytho-Siberian burials. In the published samples of the ancient Pre-Scythian population in Mongolia, haplogroups Q-YP1102, Q-YP771-YP761 and R1a-Y39884 were not found, but haplogroups R1a-Z94 and Q-YP771-BZ180 were identified [32,72,89]. The last two haplogroups were also found in representatives of the MT culture from synchronous burials in Tuva. R1a-Z94 and Q-BZ180 were also common during the Scytho-Siberian period in the representatives of the Aldy-Bel (AB) culture and the more recent Uyuk-Sagly (US) culture, which does not contradict the assumption of their connection with the Pre-Scythian population (Supplementary Table S4). One of Q-BZ180 lineages (Q-BZ180-FT421589) detected in a sample from the AB culture in Tuva was widespread in the region in Pre-Scythian times (Supplementary Table S5): in the Munkh-Khairkhan culture of Mongolia (~3600 YBP, [72]), as well as the Khirigsuur and Deer Stone cultures in Eurasian steppes (3200–2900 YBP, [72,90]).

The population that lived during the Scytho-Siberian period (the AB culture of Tuva) carried relict haplogroups R1a-YP1272, Q-BZ180, and N-Y147969, which do not occur in modern Tuvans. In the gene pools of the AB culture, which were the later stages of the US culture in Tuva and Mongolia, another R1a variant (R1a-Y39884) was common. This could indicate some kinship between representatives of the US culture that are separated by the Tannu-Ola Mountain range, which forms a natural border between Tuva and Mongolia [72] (Supplementary Table S5). The predominant location of modern R1a-Y39884 carriers in Siberia and the discovery of this branch in samples from the Scytho-Siberian period suggest that ancient R1a-Y39884 carriers already arrived in Tuva and Mongolia from Siberia in the Pre-Scythian period. R1a-Y39884 has survived through the centuries and now makes up ~8% of the Tuvan gene pool; it is typically found in Central Tuva, specifically in the Mongush and Oyun clans (Figure 7).

Summing up, two R1a-Z93 branches (R1a-FGC82884 and R1a-Z94) can be found in the ancient Scytho-Siberian and the modern population of Tuva. This supports the hypothesis that haplogroup R1a in Tuvans is not associated with migrations of European populations in historic times but reflects the long history of the autochthonous population, starting from the Andronovo culture period. In Figure 6, the clusters of ancient Siberian Scythians do not coincide with the clusters of modern Tuvans. This fact appeared to indicate that the ancient population analyzed is not the direct ancestor of modern Tuvans. Furthermore, according to our estimates, the most recent common ancestor of modern Tuvan R1a Y-STR clusters lived in a later period, 1200–400 YBP (Figure 6).

Of all Q branches, only Q-YP1102 and Q-YP771-BZ180 were found in the ancient Scytho-Siberian samples from the Eerbek River valley in Tuva (Figure 4). Unfortunately, the authors of the recent study [52] were able to identify only the ancestral haplogroup Q-L476 (Q-L53) for a warrior from a burial at Chinge-Tey. Importantly, the gene pool of the Mongolian US (Chandman) culture was not only dominated by Q-YP671, a haplogroup related to Q-YP771 from the Okunevo culture of South Siberia [32,72,91] but also included haplogroup Q-YP1102 [72]. Specimens from Chandman graves in Mongolia are essential for understanding the genetic history of South Siberia because these burials are geographically close to Tuva: the distance between the Eerbek River valley, home to the analyzed Scytho-Siberian population, and the Chandman peak in Mongolia is only 240 km. Today, haplogroup Q-YP771 occurs at low frequencies in Central Asia, China and Europe but, to our knowledge, is not found in Siberia.

So far, only branches of Q-YP1102 have been detected in modern Tuvans (Figure 4). This haplogroup, in our opinion, marks the ancient Yeniseian substrate in the modern populations of West and South Siberia. In the Scythian population, Q-YP1102 occurred only at the early stages of the US culture in Tuva and in Mongolia (Chandman [72]). This supports the hypothesis of archeologists about the migrants who gave rise to the US culture in Tuva [49]. We cautiously suggest that carriers of the Q-YP1102 branches who arrived at the Eerbek River valley around 2400 YBP originated from a population that lived downstream along the Yenisei River; perhaps they could have also inhabited other parts of Tuva. Despite the absence of Q-YP1102 in the gene pool of the late US culture [50], the branches of this haplogroup (primarily Q-YP1691) reach 20% frequencies in modern Tuvans. As in the case of haplogroup R1a, we do not have enough evidence to claim that modern Tuvans, carriers of haplogroup Q-YP1102, are direct descendants of the ancient Scytho-Siberian population in the Eerbek River.

The findings for Q-YP1102 and R1a-Y39884 should be interpreted as broad phylogenetic affinities consistent with paternal lineage continuity. However, the available data do not allow for an interpretation of the level of inheritance. Y-STR homoplasy, incomplete Y-SNP identification, a small sample size of ancient samples, and unexplored intermediate lineages may result in truly related haplotypes that appear separated in the median-joining network. Therefore, further studies of the modern and ancient populations of Southern Siberia will allow us to more accurately trace the genealogical connections between them.

## 5. Conclusions

The study demonstrates that haplogroups Q-M242 and R1a-M513, which constitute one-third of the modern Y gene pool of Tuvans, prevailed in the ancient Scytho-Siberian population in the Eerbek River at 2800–2200 YBP. More haplogroups were observed in the modern Tuvan dataset, but robust comparison of diversity is limited by unequal sampling. This could partly be due to the small area of ancient samples studied. Haplogroups R1a-YP1272, Q-BZ180 and N-Y147969 of the ancient Scythians of Tuva are rare at present and were not found in our sample set of modern Tuvans. The Q-YP1102 and R1a-Y39884 branches are found in many modern Tuvans, but our data do not clearly indicate direct inheritance. In particular, modern branch Q-YP1102-YP1691 that emerged at ~2800 YBP and split at ~2200 YBP [54] was not detected in the ancient samples, and its Y-STR cluster in modern Tuvans is very young (670 ± 130 YBP). Other haplogroups (N-M178-F4205, O-M175, C-M217 branches) appeared in Tuva, according to our assumption, after the Scytho-Siberian period [88]. The genetic history of haplogroups N-P43, which marks the Samoyedic substrate, and N-M178-Y24317, which is ethno-specific to Tuvans, Khakassians and Shors, has not been clarified yet. We hope that further studies of the ancient Tuvan gene pool will find carriers of this haplogroup who lived over 2000 years ago.

Branches of Q-YP1102 and R1a-Y39884 that arose in Siberia are now mostly found in the indigenous populations of South and West Siberia. Haplogroup Q-YP1102 marks the Yeniseian substrate in Tuvans, Khakass, Southern Altaians, Siberian Tatars and Khants. The younger haplogroup R1a-Y39884 typical of the samples from the Siberian Andronovo culture (~3800 YBP [54]) is widespread in the modern gene pool of South Siberia, specifically in Tuvans, Khakass, Shors and Northern Altaians. Analysis of the obtained findings leads us to the conclusion about broad phylogenetic affinities consistent with the paternal lineage continuity of haplogroups Q-YP1102 and R1a-Y39884. However, the resolution of Y-SNP and Y-STR genotyping and the limited sample size of ancient samples preclude a conclusion about the direct inheritance of modern Tuvan lineages from the ancient Scytho-Siberian population.

The distribution of Y-STR haplotypes among Tuvan clans sheds light on the later stages of the genetic history of Tuvans. Despite its young age, branch Q-YP1102-YP1691 occurs at 18% and is observed almost in all Tuvan clans, increasing its frequency from west to east and reaching its peak in Todzha Tuvans. All four R1a-Z93-Y39884 clusters are typical for Central Tuva and occur mainly in the Mongush and Oyun clans. And for the South and South-East regions of Tuva, where the historical bonds with Central Asian are stronger, R1a-YP1506(xF17329) and R1a-YP1543 occur at increased frequencies and mainly in the Kyrgys, Soyan, Choodu, and Khertek clans.

## Figures and Tables

**Table 1:** Characteristics of Y-STR haplotypes from ancient DNA samples found in Tuva and their distribution across different historical periods of Siberian ethnogenesis.

Period	Pre-Scythian, Late Bronze Age	Scytho-Siberian	
Archeological culture	Mongun-Taiga culture	Aldy-Bel culture	Uyuk-Sagly culture
Designation	MT	AB	US
Dating	3300–2900 YBP	2800–2500 YBP	2500–2200 YBP
Number of samples	4	16	25
Identified Y-haplogroups	R1a, Q, R1b, C	R1a (75%), Q (12.5%), N (12.5%)	R1a (32%), Q (52%), N (8%), C (4%), I (4%)
Region	Eerbek River valley, Tuva	Eerbek River valley, Tuva	Eerbek River valley, Tuva
Publications on Y-STR haplotypes	[48]	[47,48,49]	[47,49,50]

**Table 2:** Frequencies (%) of the main Y-chromosome haplogroups in the regional gene pools of Tuvans.

	Regions of Tuva						General Characteristic of Tuvan Gene Pool		
Haplogroup	Center-North	Center-South	West	South	South-East	North-East (Todzha Tuvans)	Frequency Range Between Clans	Average Frequency for 6 Regions	Average Frequency for 4 Regions [29]
Sample size	104	87	142	120	90	90	633	633	419
C-M217(xM48,M407)	3.8	2.3	1.4	17.5	10.0	1.1	0–20.8	6.2	5.5
C-M48	12.5	2.3	11.3	4.2	1.1	3.3	0–13.1	6.3	8.7
C-M407	0	2.3	0.7	4.2	17.8	0	0–16.5	3.8	1.9
D-M174	0	0	0	2.5	0	0	0–2.8	0.5	0.2
H-M69	0	1.1	0	0	0	0	0–3.2	0.2	0
I-M170	0	0	0.7	0.8	0	0	0–3.3	0.3	1.2
J-M304	0	3.4	0	0.8	2.2	0	0–6.1	1.1	0.7
N-M231(xP43,M178)	0	0	0.7	0.8	0	0	0–0.9	0.3	1.4
N-P43	19.2	27.6	33.8	23.3	8.9	21.1	15.0–37.1	23.2	24.3
N-M178	21.2	16.1	28.2	2.5	6.7	15.6	0–45.0	15.5	18.9
O-M175	0	8.0	2.1	6.7	12.2	1.1	0–13.3	4.7	7.2
Q-M242	22.1	4.6	8.5	15.0	25.6	51.1	3.0–52.2	19.9	13.9
R1a-M198	18.3	23.0	9.9	20.8	15.6	3.3	4.0–42.4	15.0	12.3
R1b-M343	1.9	9.2	2.8	0.8	0	2.2	0–12.1	2.7	3.1
R2-M124	1.0	0	0	0	0	0	0–1.0	0.2	0
Other	0	0	0	0	0	1.1	0–4.3	0.2	0.7

Note. Y-chromosome branches exceeding the 5% threshold for a polymorphism in the total sample of Tuvans are highlighted in green. Haplogroups Q and R1a are highlighted in orange. The range of frequencies between the clans is provided for 13 clans (number of samples in the dataset *n* ≥ 20).

## Data Availability

Data and materials are available in Supplementary Materials.

## Supplementary Materials

The following supporting information can be downloaded at: https://www.mdpi.com/article/10.3390/genes17080896/s1. Supplementary Figure S1: Direct comparison of the frequencies of haplogroups QM242 and R1a-M198 in 25 South Siberian and Central Asian populations. The X axis is the frequency of haplogroup Q-M242 (%), the Y axis is the frequency of haplogroup R1a-M198 (%); each graphic sign represents one population. Colored outlines mark the three groups of populations distinguished in Figure 3: high frequencies of haplogroup Q, high frequencies of haplogroup R1a, and low or medium frequencies of haplogroups Q and R1a. Legend symbols. 1—Tuvans: West (n = 142, Q = 12, R1a = 14); 2—Tuvans: Centre-North (n = 104, Q = 23, R1a = 19); 3—Tuvans: Centre-South (n = 87, Q = 4, R1a = 20); 4—Tuvans: South (n = 120, Q = 18, R1a = 25); 5—Tuvans: South-East (n = 90, Q = 23, R1a = 14); 6—Tuvans: North-East (n = 90, Q = 46, R1a = 3); 7—Tofalars (n = 50, Q = 0, R1a = 5); 8—Khakass: Kachins (n = 76, Q = 3, R1a = 5); 9—Khakass: Koibals (n = 24, Q = 14, R1a = 7); 10—Khakass: Sagay-Beltirs (n = 24, Q = 0, R1a = 13); 11—Khakass: Sagay-Biryusa (n = 38, Q = 0, R1a = 7); 12—Mountain Shors (n = 108, Q = 0, R1a = 66); 13—Northern Altaians: Chelkans (n = 65, Q = 36, R1a = 10); 14—Northern Altaians: Kumandins (n = 56, Q = 0, R1a = 13); 15—Northern Altaians: Tubalar (n = 83, Q = 18, R1a = 41); 16—Southern Altaians: Altai-Kizhi (n = 74, Q = 6, R1a = 44); 17—Southern Altaians: Telengits (n = 129, Q = 2, R1a = 58); 18—Kyrgyz (n = 294, Q = 3, R1a = 176); 19—Kazakhs (n = 366, Q = 4, R1a = 26); 20—Mongolians: West (n = 387, Q = 14, R1a = 34); 21—Mongolians: Centre (n = 310, Q = 5, R1a = 7); 22—Mongolians: East (n = 312, Q = 3, R1a = 8); 23—Buryats of Buryatia (n = 267, Q = 0, R1a = 0); 24—Buryats of the Irkutsk Region (n = 121, Q = 1, R1a = 0); 25—Buryats of Transbaikalia (n = 228, Q = 0, R1a = 2). Total sample size 3645; 235 carriers of Q-M242 and 617 carriers of R1a-M198. Supplementary Table S1: Distribution of the initial dataset by clan and districts of the Republic of Tuva; Supplementary Table S2: Detailed 37 Y-STR genotyping data; Supplementary Table S3: Y-Haplogroup minimum and maximum frequencies in Tuvan clans; Supplementary Table S4: Ancient Y-STR haplotypes from Tuva; Supplementary Table S5: Ancient Y-chromosomes; Supplementary Table S6: Modern Y-STR haplotypes from the literature.

## Abbreviations

The following abbreviations are used in this manuscript:

SNP	Single-Nucleotide Polymorphism
STR	Short Tandem Repeat
YBP	Years Before Present
TMRCA	Time to the Most Recent Common Ancestor
PCR	Polymerase Chain Reaction
ASD	Average Squared Distance
MT	Mongun-Taiga
AB	Aldy-Bel
US	Uyuk-Sagly

## Appendix A

In the Stepwise Mutational Model, every mutation changes the allele value in the Y-STR locus by one tandem repeat (step) up or down. It is hypothesized that the probability of the number of tandem repeats going up or down in such loci is ½ [92,93].

The formula for the stepwise symmetric model of Y-STR mutations is derived from Poisson distribution for one locus:

$$P\left(m\right)=\frac{\lambda^{m}}{m!}e^{-\lambda },$$

where *m* is the number of actual single-step mutations, λ=μT is the average number of actual stepwise mutations, *μ* is the mutation intensity constant (the average rate of mutations per generation), and *T* is TMRCA estimated in generations.

The problem is that we cannot see all actual mutations. In a mutating STR locus, the only thing that changes, is the number of tandem repeats. Suppose an up mutation occurs in an STR locus. This mutation increases the number of tandem repeats by 1. In the next generations, the locus may as well go through a back mutation, and the number of tandem repeats will decrease by 1. Thus, there will be no indication that the locus has gone through two mutations, because the ancestral (original) allele will still be observed in the locus. Therefore, the observed distribution of tandem repeats in the STR loci of a modern population will differ from Poisson distribution. In other words, the number of observed mutations can be lower than the number of actual mutations.

The probability of observing k single-step mutations in a locus can be described by the formula:

$$P_{obs}(\pm k)=e^{-\lambda }I_{k}(\lambda ),\;k\geq 0,$$

where $I_{k}(z)$ is the modified Bessel function of the first kind [93].

Using this probability distribution function, we can calculate the average number of observed mutations, regardless of whether they are up or down:

$$\bar{k}=2e^{-\lambda }\sum_{i=0}^{+\infty }\;iI_{i}\left(\lambda \right)=\lambda e^{-\lambda }\left(I_{0}\left(\lambda \right)+I_{1}(\lambda )\right)$$

For young clusters of Y-STR haplotypes (λ<1), a simple approximation can be applied:

$$\bar{k}=\frac{\mu T}{2}(1+e^{-\mu T}),$$

which ensures the accuracy of at least 1% for λ=μT≤0.8.

## References

[1] Serdobov N.A. (1971). The History of Formation of Tuvan People.

[2] Tatarintsev B.I. (1980). About the Some Tuvan Ethnonyms. The New Research of Tuva Archeology and Ethnogenesis of Tuvan People.

[3] Mannai-ool M.K. (2004). Tuvan People. The Origin and Formation of the Ethnos.

[4] Balanovsky O., Zhabagin M., Agdzhoyan A., Chukhryaeva M., Zaporozhchenko V., Utevska O., Highnam G., Sabitov Z., Greenspan E., Dibirova K. (2015). Deep phylogenetic analysis of haplogroup G1 provides estimates of SNP and STR mutation rates on the human Y-chromosome and reveals migrations of Iranic speakers. PLoS ONE.

[5] Balanovska E., Yusupov Y., Shalyaho R., Stepanov G., Asilgujin R., Zhabagin M., Balaganskaya O., Sultanova G., Borisova E., Daragan D. (2017). Genetic portraits of seven clans of North-Western Bashkirs: Contribution of the Finno-Ugric genetic component to the Bashkirian gene pool. Mosc. Univ. Anthropol. Bull..

[6] Balanovska E.V., Damba L.D., Agdzhoyan A.T., Zhabagin M.K., Olkova M.V., Kagazezheva Z.A., Utrivan S.A., Koshel S.M., Dybo A.V., Balanovsky O.P. (2019). The gene pool of hunter-reindeer herders of Southern Siberia: Tofalars and Todzhins. Mosc. Univ. Anthropol. Bull..

[7] Damba L.D., Balanovskaya E.V., Agdzhoyan A.T., Korotkova N.A., Olkova M.V., Utrivan S.A., Pylev V.Y., Aiyzhy E.V., Dorzhu C.M., Mongush B.B. (2019). Gene pool of three Eastern Tuvans clans according to Y-chromosome polymorphism. Mosc. Univ. Anthropol. Bull..

[8] Zhabagin M.K., Damba L.D., Korotkova N.A., Chernishenko D.N., Utrivan S.A., Pilev V.Y., Olkova M.V., Balanovska E.V., Yankovsky N.K., Balanovsky O.P. (2020). Analysis of Clan Structure of Tuvans by Y-Chromosome Markers. Russ. J. Genet..

[9] Balanovska E.V., Damba L.D., Adamov D.S., Ponomarev G.Y., Potanina A.Y., Pocheshkhova E.A. (2025). Diversity of gene pools in twelve Tuvan tribal groups (based on Y-chromosome haplogroup data). Lomonosov J. Anthropol..

[10] Karafet T.M., Zegura S.L., Posukh O., Osipova L., Bergen A., Long J., Goldman D., Klitz W., Harihara S., de Knijff P. (1999). Ancestral Asian sources of new world Y-chromosome founder haplotypes. Am. J. Hum. Genet..

[11] Stepanov V.A., Puzyrev V.P. (2000). Haplotypes of two diallelic Y chromosome loci in the indigenous and migrant populations of Siberia. Genetika.

[12] Stepanov V.A., Puzyrev V.P. (2000). Analysis of the allele frequencies of seven Y-chromosome microsatellite loci in three Tuvinian populations. Genetika.

[13] Stepanov V.A., Puzyrev V.P. (2000). Y-chromosome microsatellite haplotypes demonstrate absense of subdivision and presence of several components in the tuvinian male gene pool. Genetika.

[14] Stepanov V.A., Khitrinskaya I.Y., Puzyrev V.P. (2001). Genetic differentiation of the Tuva population with respect to the Alu-insertions. Russ. J. Genet..

[15] Wells R.S., Yuldasheva N., Ruzibakiev R., Underhill P.A., Evseeva I., Blue-Smith J., Jin L., Su B., Pitchappan R., Shanmugalakshmi S. (2001). The Eurasian heartland: A continental per spective on Y-chromosome diversity. Proc. Natl. Acad. Sci. USA.

[16] Karafet T.M., Osipova L.P., Gubina M., Posukh O.L., Zegura S.L., Hammer M.F. (2002). High Levels of Y-Chromosome Differentiation among Native Siberian Populations and the Genetic Signature of a Boreal Hunter-Gatherer Way of Life. Hum. Biol..

[17] Lell J.T., Sukernik R.I., Starikovskaya Y.B., Su B., Jin L., Schurr T.G., Underhill P.A., Wallace D.C. (2002). The dual origin and Siberian affinities of Native American Y chromosomes. Am. J. Hum. Genet..

[18] Derenko M.V., Malyarchuk B.A., Denisova G.A., Dorzhu C.M., Karamchakova O.N., Luzina F.A., Lotosh E.A., Dambueva I.K., Ondar U.N., Zakharov I.A. (2002). Polymorphism of the Y-chromosome diallelic loci in ethnic groups of the Altai-Sayan Region. Russ. J. Genet..

[19] Zerjal T., Xue Y., Bertorelle G., Wells R.S., Bao W., Zhu S., Qamar R., Ayub Q., Mohyuddin A., Fu S. (2003). The genetic legacy of the Mongols. Am. J. Hum. Genet..

[20] Tambets K., Rootsi S., Kivisild T., Help H., Serk P., Loogvali E.L., Tolk H.-V., Reidla M., Metspalu E., Pliss L. (2004). The Western and Eastern Roots of the Saami—The Story of Genetic “Outliers” Told by Mitochondrial DNA and Y Chromosomes. Am. J. Hum. Genet..

[21] Pakendorf B., Novgorodov I.N., Osakovskij V.L., Danilova A.P., Protod’jakonov A.P., Stoneking M. (2006). Investigating the effects of prehistoric migrations in Siberia: Genetic variation and the origins of Yakuts. Hum. Genet..

[22] Derenko M.V., Malyarchuk B.A., Wozniak M., Dambuveva I.K., Dorzhu C.M., Luzina F.A., Lee H.K., Miscicka-Sliwka D., Zakharov I.A. (2006). The diversity of Y-chromosome lineages in indigenous population of South Siberia. Dokl. Biol. Sci..

[23] Stepanov V.A., Kharkov V.N., Puzyrev V.P. (2006). Evolution and phylogeography of human Y-chromosomal lineages. Vavilov J. Genet. Breed..

[24] Derenko M., Malyarchuk B., Denisova G., Wozniak M., Grzybowski T., Dambueva I., Zakharov I. (2007). Y-chromosome haplogroup N dispersals from south Siberia to Europe. J. Hum. Genet..

[25] Kharkov V.N., Stepanov V.A., Medvedeva O.F., Spiridonova M.G., Puzyrev V.P., Voevoda N.I., Puzyrev V.P. (2007). Gene pool differences between Northern and Southern Altaians inferred from the data on Y-chromosomal haplogroups. Russ. J. Genet..

[26] Kharkov V.N., Medvedeva O.F., Luzina F.A., Kolbasko A.V., Gafarov N.I., Puzyrev V.P., Stepanov V.A. (2009). Comparative Characteristics of the Gene Pool of Teleuts Inferred from Y-Chromosomal Marker Data. Russ. J. Genet..

[27] Balaganskaya O.A., Lavryashina M.B., Kuznetchova M.A., Romanov A.G., Dibirova K.D., Frolova S.A., Kuznetsova A.A., Zakharova T.A., Baranova E.E., Teuchezh E.E. (2011). Gene pool of the Altai ethnic groups (from Russia, Kazakhstan, and Mongolia) analyzed by the Y chromosomal markers. Mosc. Univ. Anthropol. Bull..

[28] Dulik M.C., Zhadanov S.I., Osipova L.P., Askapuli A., Gau L., Gokcumen O., Rubinstein S., Schurr T.G. (2012). Mitochondrial DNA and Y chromosome variation provides evidence for a recent common ancestry between Native Americans and Indigenous Altaians. Am. J. Hum. Genet..

[29] Kharkov V.N., Khamina K.V., Medvedeva O.F., Simonova K.V., Khitrinskaya I.Y., Stepanov V.A. (2013). Gene-pool structure of Tuvans inferred from Y-chromosome mark er data. Russ. J. Genet..

[30] Ilumäe A.M., Reidla M., Chukhryaeva M., Järve M., Post H., Karmin M., Saag L., Agdzhoyan A., Kushniarevich A., Litvinov S. (2016). Human Y chromosome haplogroup N: A non-trivial time-resolved phylogeography that cuts across language families. Am. J. Hum. Genet..

[31] Damba L.D., Balanovskaya E.V., Zhabagin M.K., Yusupov Y.M., Bogunov Y.V., Sabitov Z.M., Agdzhoyan A.T., Korotkova N.A., Lavryashina M.B., Mongush B.B. (2018). Estimating the impact of Mongol expansion on gene pool of Tuvans. Vavilov J. Genet. Breed..

[32] Jeong C., Wang K., Wilkin S., Taylor W.T.T., Miller B.K., Bemmann J.H., Stahl R., Chiovelli C., Knolle F., Ulziibayar S. (2020). A Dynamic 6000-Year Genetic History of Eurasia’s Eastern Steppe. Cell.

[33] Agdzhoyan A.T., Damba L.D., Gurianov V.M., Zaporozhchenko V.V., Balanovsky O.P. (2021). Phylogenetic Analysis of the South Siberian Haplogroup Q-YP1102 from Y-SNP and Y-STR-Data on Tuvinian and Surrounding Populations. Russ. J. Genet..

[34] Agdzhoyan A.T., Damba L.D., Zaporozhchenko V.V., Balanovsky O.P. (2021). In addressing the question about the Samoyedic substrate in the South Siberian populations: The phylogeography of Y-chromosome haplogroup N-L666. Mosc. Univ. Anthropol. Bull..

[35] Damba L.D., Zaporozhchenko V.V., Balanovsky O.P., Balanovska E.V. (2021). Phylogenetic analysis of Y-chromosomal haplogroup C2a1a2a2a2-SK1066 in the general pool of Tuvan general groups in the context of Central Asian populations. Vestn. Tuvan State Univ..

[36] Damba L.D., Aiygy E.V., Balanovsky O.P., Markina N.V., Zhabagin M.K., Balanovskaya E.V. (2022). The Central Asian component in the gene pool of the Tuvan tribal group Mongush: On the question of the Mongolian contribution to the ethnogenesis of the Tuvans. Mosc. Univ. Anthropol. Bull. Anthropol..

[37] Stepanov V.A., Kolesnikov N.A., Valikhova L.V., Zarubin A.A., Khitrinskaya I.Y., Kharkov V.N. (2023). Structure and origin of Tuvan gene pool according to autosome SNP and Y-chromosome haplogroups. Vavilovskii Zhurnal Genet. I Sel. Vavilov J. Genet. Breed..

[38] Damba L.D., Pylev V.Y., Ponomarev G.Y., Balanovska E.V. (2024). Association of the structure of the gene pool of the Tuvan tribal group Mongush according to data on Y-chromosome polymorphism. Med. Genet..

[39] Kharkov V.N., Urmanchieva A.Y., Kolesnikov N.A., Zarubin A.A., Valikhova L.V., Khitrinskaya I.Y., Gruntov I.A., Egorov I.M., Lemskaya V.M., Dybo A.V. (2024). Population genetics and comparative linguistics: Phylogeny and phylogeography of haplogroup N1a2b and the linguistic prehistory of the Samoyed peoples. J. Lang. Relatsh..

[40] Keyser C., Bouakaze C., Crubézy E., Nikolaev V.G., Montagnon D., Reis T., Ludes B. (2009). Ancient DNA provides new insights into the history of south Siberian Kurgan people. Hum. Genet..

[41] Kolesnikova S.Y., Kharkov V.N., Valikhova L.V. (2022). To a question of the Yeniseian ethnogenesis. Vestn. Tomsk. Gos. Univ. Istor.—Tomsk. State Univ. J. Hist..

[42] Zeng T.C., Vyazov L.A., Kim A., Flegontov P., Sirak K., Maier R., Lazaridis I., Akbari A., Frachetti M., Tishkin A.A. (2025). Ancient DNA reveals the prehistory of the Uralic and Yeniseian peoples. Nature.

[43] Damgaard P.D.B., Martiniano R., Kamm J., Moreno-Mayar J.V., Kroonen G., Peyrot M., Barjamovic G., Rasmussen S., Zacho C., Baimukhanov N. (2018). The first horse herders and the impact of early Bronze Age steppe expansions into Asia. Science.

[44] Flegontov P., Altınışık N.E., Changmai P., Rohland N., Mallick S., Adamski N., Bolnick D.A., Broomandkhoshbacht N., Candilio F., Culleton B.J. (2019). Palaeo-Eskimo genetic ancestry and the peopling of Chukotka and North America. Nature.

[45] Kılınç G.M., Kashuba N., Koptekin D., Bergfeldt N., Dönertaş H.M., Rodríguez-Varela R., Shergin D., Ivanov G., Kichigin D., Pestereva K. (2021). Human population dynamics and Yersinia pestis in ancient northeast Asia. Sci. Adv..

[46] Unterländer M., Palstra F., Lazaridis I., Pilipenko A., Hofmanová Z., Groß M., Sell C., Blöcher J., Kirsanow K., Rohland N. (2017). Ancestry and demography and descendants of Iron Age nomads of the Eurasian Steppe. Nat. Commun..

[47] Mary L., Zvénigorosky V., Kovalev A., Gonzalez A., Fausser J.-L., Jagorel F., Kilunovskaya M., Semenov V., Crubézy E., Ludes B. (2019). Genetic kinship and admixture in Iron Age Scytho-Siberians. Hum. Genet..

[48] Kilunovskaya M.E., Alborova I.E., Busova V.S., Brown S., Lazarevskaya N.A., Mustafin K.K., Semenov V.A., Smirnov N.Y., Uchaneva E.N., Khavrin S.V. (2022). A Comprehensive Study of the Monuments of the End of 2nd Millennium—Beginning of 1st Millennium BC in the Valley of the River Eerbek (Central Tuva). Sib. Istor. Issled.—Sib. Hist. Res..

[49] Kilunovskaya M.E., Alborova I.A., Busova V.S., Semenov V.A., Mustafin K.K., Uchaneva E.N. (2023). Archaeological sites of the mid-1st millennium in the Urochishche Eki-Ottug (Central Tuva): From typology and chronology to anthropology and genetics. Arkheologicheskie Vesti.

[50] Kilunovskaya M.E., Alborova I.E., Anikeeva O.V., Brown S., Busova V.S., Kozhukhova E.I., Mustafin K.K., Semenov V.A., Uchaneva E.N. (2024). Reconstructing Ethnocultural Processes in the Sausken Valley (Tuva) during the Later Half of the 1st Millennium BC. Ufa Archaeol. Her..

[51] McColl H., Kroonen G., Moreno-Mayar V.J., Seersholm F.V., Scorrano G., Pinotti T., Vimala T., Sindbæk S., Ethelberg P., Fyfe R. (2024). Steppe Ancestry in western Eurasia and the spread of the Germanic Languages. bioRxiv.

[52] Nedoluzhko A., Vergasova E., Sharko F., Agapitova N., Kharitonov D., Sukhanova X., Pushkina O., Pankova S., Slobodova N., Boulygina E. (2025). Ancient DNA analysis of elite nomadic warrior from Chinge-Tey I funerary commemorative complex in the “Valley of the Kings”, Tuva. BMC Genom..

[53] Zvenigorosky V., Cervel M., Gonzalez A., Zazzo A., Fausser J.-L., Bayarkhuu N., Bernard V., Joly D., Ludes B., Turbat T. A Family Portrait in Death: Burial Decisions and Social Structure at the Iron Age Saghil Site (Mongolia). https://ssrn.com/abstract=6788851.

[54] (2025). YFull YTree v13.07.00. https://www.yfull.com/tree/.

[55] Balanovsky O., Rootsi S., Pshenichnov A., Kivisild T., Churnosov M., Evseeva I., Pocheshkhova E., Boldyreva M., Yankovsky N., Balanovska E. (2008). Two sources of the Russian patrilineal heritage in their Eurasian context. Am. J. Hum. Genet..

[56] Bandelt H.J., Forster P., Röhl A. (1999). Median-joining networks for inferring intraspecific phylogenies. Mol. Biol. Evol..

[57] Zhivotovsky L.A., Underhill P.A., Cinnioğlu C., Kayser M., Morar B., Kivisild T., Scozzari R., Cruciani F., Destro-Bisol G., Spedini G. (2004). The effective mutation rate at Y chromosome short tandem repeats, with application to human population-divergence time. Am. J. Hum. Genet..

[58] Willuweit S., Roewer L. (2015). The new Y Chromosome Haplotype Reference Database. Forensic Sci. Int. Genet..

[59] Ballantyne K.N., Goedbloed M., Fang R., Schaap O., Lao O., Wollstein A., Choi Y., van Duijn K., Vermeulen M., Brauer S. (2010). Mutability of Y-Chromosomal Microsatellites: Rates, Characteristics, Molecular Bases, and Forensic Implications. Am. J. Hum. Genet..

[60] Fenner J. (2005). Cross-cultural estimation of the human generation interval for use in genetics-based population divergence studies. Am. J. Phys. Anthropol..

[61] Adamov D.S., Alekseev A.N., Fedorova S.A. (2024). Dating the Time to the Most Recent Common Ancestor of the Sakha (Yakuts) with Y chromosomal haplogroup N3a2-M1982: New ethnogenetic reconstructions. Yakut Med. J..

[62] Gyuris B., Vyazov L., Türk A., Flegontov P., Szeifert B., Langó P., Mende B.G., Csáky V., Chizhevskiy A.A., Gazimzyanov I.R. (2025). Long shared haplotypes identify the southern Urals as a primary source for the 10th-century Hungarians. Cell.

[63] Grugni V., Raveane A., Ongaro L., Battaglia V., Trombetta B., Colombo G., Capodiferro M.R., Olivieri A., Achilli A., Perego U.A. (2019). Analysis of the human Y-chromosome haplogroup Q characterizes ancient population movements in Eurasia and the Americas. BMC Biol..

[64] Isakova Z., Bukayev A., Irsaliev M., Sabitov Z., Zhabagin M. (2025). Population data of 23 Y chromosome STR loci for Kyrgyz population from Kyrgyzstan. Data Brief.

[65] Zhabagin M., Sarkytbayeva A., Tazhigulova I., Yerezhepov D., Li S., Akilzhanov R., Yeralinov A., Sabitov Z., Akilzhanova A. (2019). Development of the Kazakhstan Y-chromosome haplotype reference database: Analysis of 27 Y-STR in Kazakh population. Int. J. Leg. Med..

[66] Ashirbekov Y., Nogay A., Abaildayev A., Zhunussova A., Sabitov Z., Zhabagin M. (2023). Genetic polymorphism of 27 Y-STR loci in Kazakh populations from Eastern Kazakhstan. Ann. Hum. Biol..

[67] Ashirbekov Y., Seidualy M., Abaildayev A., Maxutova A., Zhunussova A., Akilzhanova A., Sharipov K., Sabitov Z., Zhabagin M. (2023). Genetic polymorphism of Y-chromosome in Kazakh populations from Southern Kazakhstan. BMC Genom..

[68] Valikhova L.V., Kharkov V.N., Zarubin A.A., Kolesnikov N.A., Svarovskaya M.G., Khitrinskaya I.Y., Shtygasheva O.V., Volkov V.G., Stepanov V.A. (2022). Genetic Interrelation of the Chulym Turks with Khakass and Kets according to Autosomal SNP Data and Y-Chromosome Haplogroups. Russ. J. Genet..

[69] Tambets K., Yunusbayev B., Hudjashov G., Ilumäe A.-M., Rootsi S., Honkola T., Vesakoski O., Atkinson Q., Skoglund P., Kushniarevich A. (2018). Genes reveal traces of common recent demographic history for most of the Uralic-speaking populations. Genome Biol..

[70] Karmin M., Saag L., Vicente M., Sayres M.A.W., Järve M., Talas U.G., Rootsi S., Ilumäe A.-M., Mägi R., Mitt M. (2015). A recent bottleneck of Y chromosome diversity coincides with a global change in culture. Genome Res..

[71] Ning C., Wang C.-C., Gao S., Yang Y., Zhang X., Wu X., Zhang F., Nie Z., Tang Y., Robbeets M. (2019). Ancient Genomes Reveal Yamnaya-Related Ancestry and a Potential Source of Indo-European Speakers in Iron Age Tianshan. Curr. Biol..

[72] Wang C.-C., Yeh H.-Y., Popov A.N., Zhang H.-Q., Matsumura H., Sirak K., Cheronet O., Kovalev A., Rohland N., Kim A.M. (2021). Genomic insights into the formation of human populations in East Asia. Nature.

[73] Kumar V., Wang W., Zhang J., Wang Y., Ruan Q., Yu J., Wu X., Hu X., Wu X., Guo W. (2022). Bronze and Iron Age population movements underlie Xinjiang population history. Science.

[74] Lee J., Miller B.K., Bayarsaikhan J., Johannesson E., Miller A.V., Warinner C., Jeong C. (2023). Genetic population structure of the Xiongnu Empire at imperial and local scales. Sci. Adv..

[75] Liu W.-J., Pu H.-W., Yang C.-H., Meng H.-T., Zhang Y.-D., Zhang L.-P., Yan J., Wang H., Ren J., Sun J. (2015). 24 Y-chromosomal STR haplotypic polymorphisms for Chinese Uygur ethnic group and its phylogenic analysis with other Chinese groups. Electrophoresis.

[76] Bian Y., Zhang S., Zhou W., Zhao Q., Siqintuya, Zhu R., Wang Z., Gao Y., Hong J., Lu D. (2016). Analysis of genetic admixture in Uyghur using the 26 Y-STR loci system. Sci. Rep..

[77] Underhill P.A., Poznik G.D., Rootsi S., Järve M., Lin A.A., Wang J., Passarelli B., Kanbar J., Myres N.M., King R.J. (2015). The phylogenetic and geographic structure of Y-chromosome haplogroup R1a. Eur. J. Hum. Genet..

[78] Saag L., Vasilyev S.V., Varul L., Kosorukova N.V., Gerasimov D.V., Oshibkina S.V., Griffith S.J., Solnik A., Saag L., D’atanasio E. (2021). Genetic ancestry changes in Stone to Bronze Age transition in the East European plain. Sci. Adv..

[79] Mathieson I., Lazaridis I., Rohland N., Mallick S., Patterson N., Roodenberg S.A., Harney E., Stewardson K., Fernandes D., Novak M. (2015). Genome-wide patterns of selection in 230 ancient Eurasians. Nature.

[80] Saag L., Varul L., Scheib C.L., Stenderup J., Allentoft M.E., Saag L., Pagani L., Reidla M., Tambets K., Metspalu E. (2017). Extensive Farming in Estonia Started through a Sex-Biased Migration from the Steppe. Curr. Biol..

[81] Andreeva T.V., Soshkina A.D., Gusev F.E., Malyarchuk A.B., Dotsenko G.S., Dudko N.A., Plotnikova M.Y., Kunizheva S.S., Manakhov A.D., Ustkachkintseva T.V. (2025). Genetic history of Scythia. Sci. Adv..

[82] Gnecchi-Ruscone G.A., Khussainova E., Kahbatkyzy N., Musralina L., Spyrou M.A., Bianco R.A., Radzeviciute R., Martins N.F.G., Freund C., Iksan O. (2021). Ancient genomic time transect from the Central Asian Steppe unravels the history of the Scythians. Sci. Adv..

[83] Damgaard P.d.B., Marchi N., Rasmussen S., Peyrot M., Renaud G., Korneliussen T., Moreno-Mayar J.V., Pedersen M.W., Goldberg A., Usmanova E. (2018). 137 ancient human genomes from across the Eurasian steppes. Nature.

[84] Nedoluzhko A., Pankova S., Vergasova E., Plotnikov N., Kim A., Shulpin M., Nenasheva N., Adameyko K., Poliakov A., Pogodina N. (2022). First ancient DNA analysis of mummies from the post-Scythian Oglakhty cemetery in South Siberia. Res. Sq..

[85] Song F., Song M., Luo H., Xie M., Wang X., Dai H., Hou Y. (2021). Paternal genetic structure of Kyrgyz ethnic group in China revealed by high-resolution Y-chromosome STRs and SNPs. Electrophoresis.

[86] Adamov D., Ponomarev G., Evsyukov I., Zhabagin M., Belenikin M., Antonenko A., Belov R., Balanovska E. (2025). Phylogeography and Microevolution of Y-Chromosome Haplogroup N-B482: Ancient Diffusion and Modern Relicts. Nat. Anthropol..

[87] Pilipenko A.S., Trapezov R.O., Polosmak N.V. (2015). A paleogenetic study of Pazyryk people buried at Ak-Alakha-1, the Altai Mountains. Archaeol. Ethnol. Anthropol. Eurasia.

[88] Adamov D., Damba L., Ponomarev G., Pocheshkhova E., Balanovska E. (2026). Ethnogenesis Reconstruction in the Population of South Siberia and Tuva Using Data on N-M178 and O-M175 Polymorphisms. Nat. Anthropol..

[89] Jeong C., Wilkin S., Amgalantugs T., Bouwman A.S., Taylor W.T.T., Hagan R.W., Bromage S., Tsolmon S., Trachsel C., Grossmann J. (2018). Bronze Age population dynamics and the rise of dairy pastoralism on the eastern Eurasian steppe. Proc. Natl. Acad. Sci. USA.

[90] Lee J., Brosseder U., Moon H., Stahl R., Semerau L., Gantulga J.-O., Magail J., Bemmann J., Yeruul-Erdene C., Warinner C. (2025). Slab Grave expansion disrupted long co-existence of distinct Bronze Age herders in central Mongolia. Nat. Commun..

[91] Liu L., Xie S., Li H., Wei L., Yu H. (2025). Demographic History of Ancient Okunev People and Their Kin across Eurasia: A Patrilineal Perspective. Nat. Anthropol..

[92] Walsh B. (2001). Estimating the time to the most recent common ancestor for the Y chromosome or mitochondrial DNA for a pair of individuals. Genetics.

[93] Watkins J.C. (2007). Microsatellite evolution: Markov transition functions for a suite of models. Theor. Popul. Biol..

