# Supplementary material for: The Yeniseian and Andronovo Substrates in the Gene Pools of the Indigenous Southern Siberian Populations Analyzed by Q and R1a Y-Haplogroups
Source: Genes (Basel). 2026 Jul 30;17(8):896. doi: 10.3390/genes17080896 (PMC13512732; doi:10.3390/genes17080896)
Supplement: Supplementary file 1 [file genes-17-00896-s001.zip › Supplementary Figure S1 new.pdf]

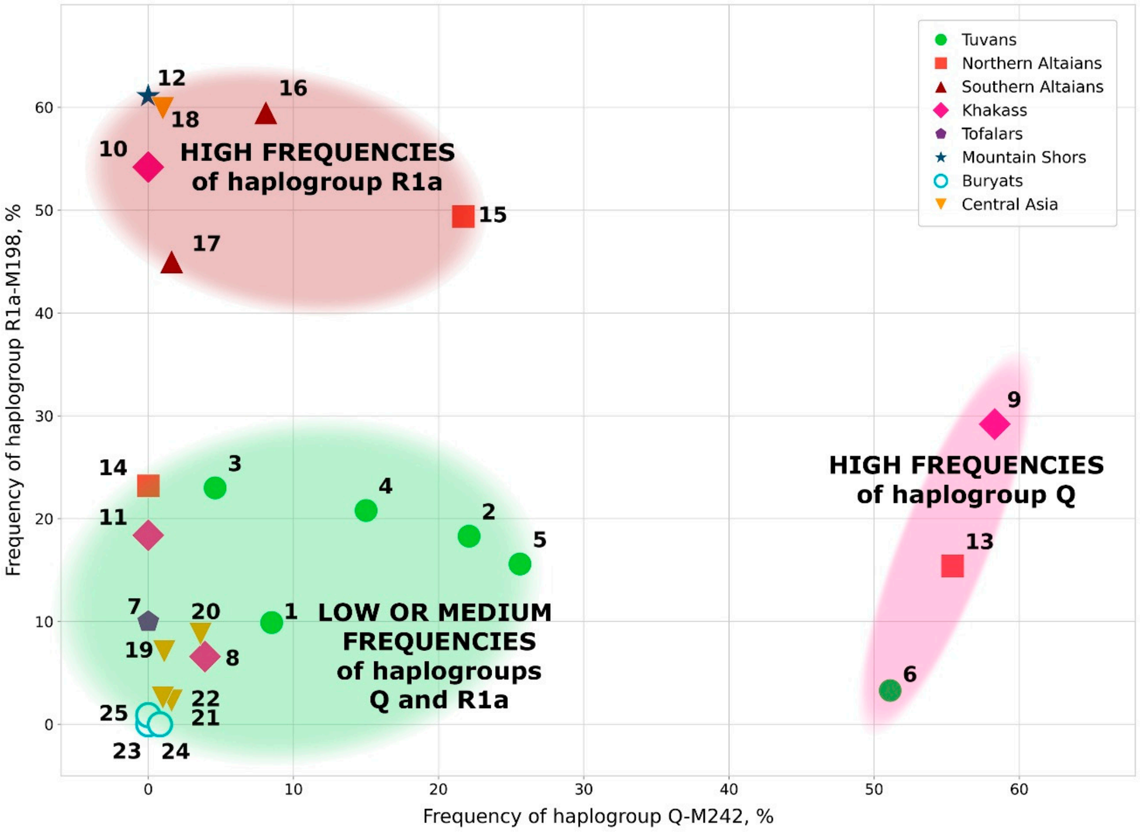

Supplementary Figure S1. Direct comparison of the frequencies of haplogroups Q-M242 and R1a-M198 in 25 South Siberian and Central Asian populations. The X axis is the frequency of haplogroup Q-M242 (%), the Y axis is the frequency of haplogroup R1a-M198 (%); each graphic sign represents one population. Colored outlines mark the three groups of populations distinguished in Figure 3: high frequencies of haplogroup Q, high frequencies of haplogroup R1a, and low or medium frequencies of haplogroups Q and R1a. Legend symbols. 1 – Tuvans: West (n = 142, Q = 12, R1a = 14); 2 – Tuvans: Centre-North (n = 104, Q = 23, R1a = 19); 3 – Tuvans: Centre-South (n = 87, Q = 4, R1a = 20); 4 – Tuvans: South (n = 120, Q = 18, R1a = 25); 5 – Tuvans: South-East (n = 90, Q = 23, R1a = 14); 6 – Tuvans: North-East (n = 90, Q = 46, R1a = 3); 7 – Tofalars (n = 50, Q = 0, R1a = 5); 8 – Khakass: Kachins (n = 76, Q = 3, R1a = 5); 9 – Khakass: Koibals (n = 24, Q = 14, R1a = 7); 10 – Khakass: Sagay-Beltirs (n = 24, Q = 0, R1a = 13); 11 – Khakass: Sagay-Biryusa (n = 38, Q = 0, R1a = 7); 12 – Mountain Shors (n = 108, Q = 0, R1a = 66); 13 – Northern Altaians: Chelkans (n = 65, Q = 36, R1a = 10); 14 – Northern Altaians: Kumandins (n = 56, Q = 0, R1a = 13); 15 – Northern Altaians: Tubalar (n = 83, Q = 18, R1a = 41); 16 – Southern Altaians: Altai-Kizhi (n = 74, Q = 6, R1a = 44); 17 – Southern Altaians: Telengits (n = 129, Q = 2, R1a = 58); 18 – Kyrgyz (n = 294, Q = 3, R1a = 176); 19 – Kazakhs (n = 366, Q = 4, R1a = 26); 20 – Mongolians: West (n = 387, Q = 14, R1a = 34); 21 – Mongolians: Centre (n = 310, Q = 5, R1a = 7); 22 – Mongolians: East (n = 312, Q = 3, R1a = 8); 23 – Buryats of Buryatia (n = 267, Q = 0, R1a = 0); 24 – Buryats of the Irkutsk Region (n = 121, Q = 1, R1a = 0); 25 – Buryats of Transbaikalia (n = 228, Q = 0, R1a = 2). Total sample size 3645; 235 carriers of Q-M242 and 617 carriers of R1a-M198.
